# BAC-Based Sequencing of Behaviorally-Relevant Genes in the Prairie Vole

**DOI:** 10.1371/journal.pone.0029345

**Published:** 2012-01-06

**Authors:** Lisa A. McGraw, Jamie K. Davis, Pamela J. Thomas, NISC Comparative Sequencing Program, Larry J. Young, James W. Thomas

**Affiliations:** 1 Center for Translational Social Neuroscience and Yerkes National Primate Research Center, Emory University, Atlanta, Georgia, United States of America; 2 Department of Human Genetics, Emory University School of Medicine, Atlanta, Georgia, United States of America; 3 Genome Technology Branch and NIH Intramural Sequencing Center, National Human Genome Research Institute, National Institutes of Health, Bethesda, Maryland, United States of America; 4 Department of Psychiatry and Behavioral Sciences, Emory University School of Medicine, Atlanta, Georgia, United States of America; Auburn University, United States of America

## Abstract

The prairie vole (*Microtus ochrogaster*) is an important model organism for the study of social behavior, yet our ability to correlate genes and behavior in this species has been limited due to a lack of genetic and genomic resources. Here we report the BAC-based targeted sequencing of behaviorally-relevant genes and flanking regions in the prairie vole. A total of 6.4 Mb of non-redundant or haplotype-specific sequence assemblies were generated that span the partial or complete sequence of 21 behaviorally-relevant genes as well as an additional 55 flanking genes. Estimates of nucleotide diversity from 13 loci based on alignments of 1.7 Mb of haplotype-specific assemblies revealed an average pair-wise heterozygosity (8.4×10^−3^). Comparative analyses of the prairie vole proteins encoded by the behaviorally-relevant genes identified >100 substitutions specific to the prairie vole lineage. Finally, our sequencing data indicate that a duplication of the prairie vole *AVPR1A* locus likely originated from a recent segmental duplication spanning a minimum of 105 kb. In summary, the results of our study provide the genomic resources necessary for the molecular and genetic characterization of a high-priority set of candidate genes for regulating social behavior in the prairie vole.

## Introduction

The prairie vole (*Microtus ochrogaster*) is a North American Microtine rodent that has become a premier animal model for the study of social behavior and has proven useful for discovering gene-brain-behavior relationships [1], [2]. Unlike the majority of mammalian species, prairie voles are highly social, often form lifelong partnerships with their mates (pair bonds) and both parents take part in rearing offspring [3]. Contrary to the socially monogamous prairie vole, other closely related Microtine vole species (i.e. *M. montanus* and *M. pennsylvanicus*) are promiscuous, largely asocial, do not form pair bonds and only females contribute to offspring care [4]. The unique differences in the social repertoires of these species has allowed for comparative studies that have led to substantial contributions to our understanding of the neural and molecular circuitry involved in behaviors such as social attachment, parental behavior, addictive behavior, effects of early life experience and social influences on physiological traits [1]. Delineating genomic characteristics that potentially differentiate the social prairie voles from other asocial rodent species may therefore provide important insights as to how the genome contributes to gene expression patterns in the brain and ultimately to both between- and within-species variation in behaviors. Further, the genetic and neurobiological mechanisms discovered to be regulating prairie vole social behavior have also been found to contribute to human social cognition (reviewed in [5]).

To date, of brain-expressed genes that contribute to the behavioral diversity in prairie voles, DNA sequence resources for studying *cis*-regulatory and/or transcriptional profiles are available for only the arginine vasopressin receptor 1a (*Avpr1a*) [6], the oxytocin receptor (*Oxt*, [7]), arginine vasopressin (*Avp*, GenBank Ac# DQ269208) and estrogen receptor-α (*Esr1*, [8]). Thus, detailed genetic and molecular studies focused on behaviorally-relevant genes in the prairie vole, like those that have associated differential distribution of AVPR1A in the brain with affiliative behavior (reviewed in [4]), are currently limited by a lack of gene and genomic sequences for this species. In this study, we selected and fully sequenced BAC clones containing 21 brain-expressed genes falling into five functional classes that are known to or likely to play a role in affiliative behavior (Table 1).

**Table 1:** List of 21 behaviorally-relevant genes.

Classification	Gene Name	Symbol
Neurohypophysial peptide system(social recognition, social information processing)	oxytocin	*Oxt*
	oxytocin receptor	*Oxtr*
	vasopressin	*Avp*
	vasopressin receptor	*Avpr1a*
Dopamine system(reward, reinforcement learning)	D1 dopamine receptor	*Drd1a*
	D2 dopamine receptor	*Drd2*
	dopamine transporter	*Slc6a3*
	norepinephrine transporter	*Slc6a2*
	tyrosine hydroxylase	*Th*
Stress axis	corticotropin releasing factor	*Crf*
	urocortin	*Ucn*
	urocortin II	*Ucn2*
	urocortin III	*Ucn3*
	CRF1 receptor	*Crf1*
	CRF2 receptor	*Crf2*
	glucocorticoid receptor	*Nr3c1*
Sex steroid receptors	estrogen receptor α	*Esr1*
	estrogen receptor β	*Esr2*
	androgen receptor	*Ar*
Synaptic plasticity	brain-derived neurotrophic factor	*Bdnf*
	neurotrophic tyrosine kinase receptor 2	*Ntkr2*

The neurohypophysial peptides, oxytocin (OXT) and vasopressin (AVP) along with their receptors, OXTR and AVPR1A, respectively, have long been known to regulate species-specific social behaviors including pair bonding, parental care, social recognition and aggression by acting within the reward circuitry regions of the brain (reviewed in [2]). Although there is little difference in the distribution of these receptors between sexes, pharmacological and transgenic manipulations have demonstrated that OXTR within the nucleus accumbens plays an important role in pair bonding in females, while AVPR1A within the ventral pallidum and lateral septum contributes to pair bond formation in males (reviewed in [4]). Like the neurohypophysial peptides and their receptors, the dopaminergic system, acting primarily within the nucleus accumbens, also plays a role in pair bonding in prairie voles. While D2 receptors (DRD2) are essential in the formation of pair bonds in males, D1 receptors (DRD1A) appear to be inhibitory [9], [10]. In voles and other species, other genes within the dopaminergic system also contribute to aspects of pair bonding such as learning and memory, parental behavior, sexual behavior, social choice and olfaction (reviewed in [2]). The hypothalamic-pituitary-adrenal (HPA) axis which plays a prominent role in the stress response has also been implicated in social bond formation in prairie voles. Corticotrophin releasing (CRHR) receptors within the nucleus accumbens facilitate pair bonding in males [11] and when a male loses his partner, CRHR receptors facilitate passive stress-coping behavior much akin to depressive behavior in our own species [12]. The effects are mediated by both CRHR1 and CRHR2 receptors, and the ligands that are potentially involved in this process are CRH, and the urocortins (UCN, UCN2, and UCN3). Sex steroid hormones are also known to contribute to the expression of affiliative behaviors. For example, in prairie voles, social affiliation is influenced by estrogen receptor alpha (ESR1) within the amygdala and the bed nucleus of the striata terminalis and by estrogen receptor beta (ESR2) within the paraventricular nucleus of the hypothalamus [13], [14], [15]. Finally, while genes involved in synaptic plasticity have not been directly implicated in affiliative behaviors within prairie voles, there is substantial potential for these genes to regulate aspects of social learning based on social experiences [16]. For example, when BDNF is knocked-down in the nucleus accumbens of mice, males can be rescued from developing an aversion to social contact after experiencing long bouts of aggression from another animal [17].

Here, we report the targeted bacterial artificial chromosome (BAC)-based sequencing and accompanying analyses of the 21 behaviorally-relevant genes and flanking regions in the prairie vole.

## Materials and Methods

### BAC sequencing, assembly and annotation

Targeted BAC-based sequencing was used to assemble 6.4 Mb of non-redundant or haplotype-specific sequence from 22 chromosomal segments that contain or immediately flank 21 behaviorally-relevant genes (Table 2). In addition to the targeted genes of interest, 55 flanking genes and a single microRNA were at least partially spanned by the sequence assemblies. With the exception of *ANKK1*, which we predict may be a pseudogene in the prairie vole, and the absence of a prairie vole ortholog of *Calm5*, the gene order, orientation and content was the same in the prairie vole as that observed in the mouse (data not shown).

**Table 2:** Summary of BAC sequencing.

Target gene(s)	Other genes	Sequence* (kb)
*Ar* **	-	234
*Avpr1a*	-	140
*Avpr1a* truncated	-	149
*Bdnf*	-	141
*Crh*	*Dnajc5b, Trim55* **	208 (155)
*Crhr1* **	*4933407p14rik, Mapt, 1700081l11rik* **	150 (161)
*Crhr2*	*Nod1* **, *Ggct* **, *Gars* **, *Inmt, Fam188b*	253
*Drd1A*	*Sfxn1, Hrh2* **	206 (182)
*Drd2*	*Ankk1, Tct12* **,*Ncam* **	282 (156)
*Esr1* **	*Syne1* **	303 (442)
*Esr2* **	*Syne2* **, *Tex21* **	106 (177)
*Mc4r*	-	141
*Nr3c1*	*Arhgap26* **	169 (170)
*Ntrk2* **	-	166
*Oxt, Avp*	*Ptpra, Mrps26, Ubox5, Fastkd5*	160 (145)
*Oxtr*	*Lmcd1* **, *D630042p16rik, Cav3* **, *Rad18*	176 (309)
*Slc6a2*	*Lpcat2* **, *Capns2* **, *Gm4976, Es1, Ces3* **	213 (137)
*Slc6a3*	*Lpcat1* **, *Clptm1l* **, *Tert* **, *Slc6a18, Slc6a19*	114 (148)
*Th*	-	86
*Ucn*	*Slc5a6, 0610007c21rik, Cad, Slc30a3, Dnajc5g* **, *Trim54*	154
*Ucn2* ***	*Ip6k2, Nckipsd, Celsr3, Slc26a6, Tmem89, Uqcrc1, Col7a1* **, *Mir711*	155 (154)
*Ucn3*	*Calml3, Calm4, Net1, Tubal3* **	158 (178)

*Size of the alternative haplotype assembly is shown in parentheses.

**Partial sequence.

***Sequence assembly is adjacent to the target gene and does not include the target gene itself.

Prairie vole BACs from the CHORI-232 library were selected for sequencing based on probe-content and restriction-enzyme fingerprint contigs constructed from clones isolated from the targeted regions of interest [18], [19]. When possible, aligned BAC-end sequences were used to select pairs of clones from the autosomal loci that represented the two alternative haplotypes present in the library using the strategy described in [20]. Individual BAC clones were either Sanger shotgun sequenced and assembled as described in [21], or pooled and shotgun sequenced using Roche 454 single-end reads. Note that the two clones pooled and sequenced using the Roche 454 platform were from different target regions and that the haplotype-specific assemblies were restricted to the Sanger sequencing of individual clones. Multi-BAC assemblies were generated from clones representing the same haplotype. Genes were annotated primarily based on alignments between mouse cDNAs and the prairie vole genomic sequence, and when available prairie vole cDNAs, using Spidey [22]. The gene annotation is available in the GenBank records listed in Table S1.

### Sequence alignments and identification of genetic variation

Genomic sequence assemblies representing alternative haplotypes were aligned with blastz [23] and used as the basis to identify SNPs and indels. Prior to the identification of SNPs, the alignments were masked to exclude low quality sites (phred score <50) as well as simple and low complexity sequence. All the identified SNPs have been deposited in dbSNP. Prairie vole protein coding regions representing the alternative haplotypes were aligned with ClustalX [24] excluding codons with one or more site with a phred score <50. Non-synonymous and synonymous SNPs were identified using PAML [25]. Amino acid sequences were also aligned with ClustalX. Orthologous proteins from other species were downloaded from GenBank or publicly available genome assemblies and are provided in File S1. Amino acid replacements unique to the prairie vole lineage were inferred using simple parsimony and represent a conservative number of changes that occurred in the prairie vole lineage. Radical amino acid substitutions were defined as those that changed at least two out of the three properties for the amino acids outlined in [26], i.e., charge, polarity, and polarity/volume, whereas conservative amino acid substitutions resulted in a change in at most one of those properties.

## Results and Discussion

### SNP and indel frequency

In order to survey the frequency and type of genetic variation present in the individual from which the BAC library was constructed, we aligned the genomic sequence assemblies derived from BAC clones representing alternative haplotypes (see Table 2 and Table 3). Pair-wise heterozygosity (π) based on single-nucleotide polymorphisms (SNPs) at the 13 sampled loci ranged from 3.6–11.0×10^−3^ with the average being 8.4×10^−3^. Insertions and deletions (indels) polymorphisms were on the order of 5-fold less abundant than the SNPs (Table 3). Similar to what has been observed in other mammals (for example see [27]), the indel length distribution was heavily skewed toward the smaller size range with 1-bp indels being the most common.

**Table 3:** Nucleotide diversity between alternative haplotypes.

Region*	Aligned sites**	SNPs	π(×10^−3^)	Indels	Indel size range
*Crh*	138,103	1,518	11.0	278	1–3,054 bp
*Crhr1*	72,493	604	8.3	108	1–3,066 bp
*Drd1A*	117,457	1,156	9.8	235	1–773 bp
*Drd2*	132,468	1,313	9.9	192	1–6,070 bp
*Esr1*	265,109	2,665	10.1	503	1–13,667 bp
*Esr2*	56,409	476	8.4	100	1–1,069
*Nr3c1*	159,447	934	5.9	219	1–680 bp
*Oxt, Avp*	138,034	931	6.7	271	1–1,091 bp
*Oxtr*	151,640	1,353	8.9	256	1–3,241 bp
*Slc6a2*	127,323	1,288	10.1	206	1–4,188 bp
*Slc6a3*	100,371	745	7.4	142	1–1,681 bp
*Ucn2*	144,027	518	3.6	119	1–338 bp
*Ucn3*	145,967	1,198	8.2	231	1–1,776 bp
Total	1,748,848	14,699	8.4	2,860	1–13,667 bp

π represents the pair-wise heterozygosity (substitutions/site) between the alternative haplotypes.

*Regions are referred to by the target gene of interest.

**Represents aligned sites after excluding simple and low complexity sequence, and sites with low sequence quality.

Genetic diversity tends to vary across a genome and is influenced by a number of factors including local recombination rates, the history of the population, and natural selection (reviewed in [28]). Thus, though sampling bias, both in term of the individuals included in a study and position in the genome, can have a strong effect on estimates of π, it is nonetheless of interest to compare the estimate of π we observed in the prairie vole to those reported for other mammals. For example, in three other rodents [the field vole (*Microtus agrestis*), wild mice (*Mus musculus*), and deer mouse (*Peromyscus maniculatus*)] sequence-based estimates of π for the nuclear genome were reported to be 0.8×10^−3^, 1.3–8.2×10^−3^, and 2.9–24.1×10^−3^, respectively [29], [30], [31]. The values of π we observed in a single prairie vole of 3.6–1.1×10^−3^, average = 8.4×10^−3^, are therefore within the range previously observed in rodents, but are higher than the nucleotide diversity observed in other mammals such as the panda (1.3×10^−3^, [32]), chimpanzee (0.8–1.9×10^−3^, [33]), and humans (0.6–0.9×10^−3^, [34] and references therein). Future studies estimating the genetic diversity in prairie voles based on multiple individuals and additional loci will be needed to determine if the level of nucleotide diversity observed in this study is truly representative of the species.

### Intra- and interspecific gene and amino acid sequence comparisons

The protein coding region of the 43 prairie vole genes sequenced on both haplotypes were aligned to identify synonymous and nonsynonymous SNPs. In total we identified 201 synonymous (dS = 9.9×10^−3^) and 75 nonsynonymous (dN = 1.5×10^−3^) SNPs between the two haplotypes sampled at each locus. Within the 12 behaviorally-relevant genes that were sequenced on both haplotypes there were 39 synonymous and 6 nonsynonymous SNPs. No SNPs were observed in *Oxtr* and *Ucn3*, synonymous but no nonsynonymous SNPs were present in seven genes (*Drd1a*, *Esr1*, *Esr2*, *Nr3c1*, *Oxt*, *Slc6a2*, *Slc6a3*), and both synonymous and nonsynonymous SNPs were observed in three genes (*Avp*, *Crhr1* And *Drd2*). To evaluate the potential functional consequence of the nonsynonymous SNPs in *Avp*, *Crhr1* And *Drd2*, and to identify amino acid replacements that were specific to the prairie vole lineage in all of the behaviorally-relevant genes, we aligned the predicted prairie vole protein sequences to orthologous proteins from other rodents: mouse (*Mus musculus*), rat (*Rattus norvegicus*), and guinea pig (*Cavia porcellus*), as well as rabbit (*Oryctolagus cuniculus*) (see Methods and File S1).

**Figure 1:**
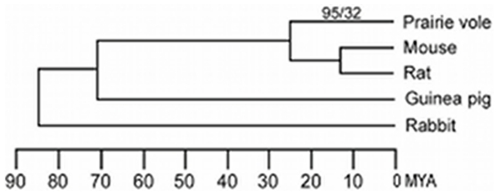
Amino acid substitutions in the prairie vole lineage. The evolutionary relationship of the prairie vole to other rodents and rabbit is illustrated as a phylogenetic tree. Divergence times are represented by the branch lengths of the tree based on [39], [40]. The numbers above the terminal branch leading to the prairie vole represent the number of conservative/radical amino acid substitutions in the behaviorally-relevant proteins that were inferred by parsimony to have occurred in that lineage and unique to the prairie vole. MYA refers to millions of years ago.

The proteins encoded by the behaviorally-relevant prairie vole genes (n = 21) were on average 93% identical (range of 84–99%) to their mouse/rat orthologs. A total of 127 unique amino acid replacements in these proteins could be assigned by parsimony to the prairie vole lineage, of which 32 were classified as radical substitutions (Fig. 1 and Table S2). The potential functional impact of the nonsynonymous SNPs in *Avp*, *Crhr1* And *Drd2* was predicted using evolutionary conservation using the program SIFT [35]. Based on this metric three of the nonsynonymous changes were predicted to affect protein function while the remaining changes were predicted to be tolerated (Table S3).

The prairie vole is considered a valuable rodent model for social behavior due to the phenotypes observed in this species that are uncommon in other rodents, such as pair-boding [1]. While differential distribution of AVPR1A in the brain has been correlated with variation in social behavior [36], lineage-specific changes that alter the regulation or proteins of other genes related to social behavior that distinguish the prairie vole from other rodents may also be functionally relevant. We therefore consider the >100 amino acid changes in proteins relevant to behavior we identified in the prairie vole lineage candidates for altering the activity of these proteins. However, since the prairie vole lineage has been evolving independently from the other rodent lineages for at least 25 million years, we anticipate that most of these lineage specific changes may have accumulated by chance and will not be functionally relevant. Future comparative studies will be needed to determine if in fact the prairie vole proteins do exhibit any differences in activity compared to other rodents and which specific changes are responsible for such functional alterations.

### Segmental duplication of the Avpr1a locus

Previous cloning and sequencing efforts of the prairie vole *Avpr1a* gene detected the presence of a duplicate copy that encoded a truncated protein [6]. To gain further insight into this duplication, we sequenced BAC clones containing either the functional and truncated prairie vole *Avpr1a* loci (Table 1). Alignment of the resulting sequences revealed a duplication of ≥105 kb spanning the *Avpr1a* loci and flanking regions. The divergence between the duplicons was 0.0177+/−0.0004 substitutions/site (87,614 sites, Kimura 2-parameter distance [37]), suggesting the duplication likely occurred relatively recently. As was reported previously [6], the truncated *Avpr1a* locus included a ∼700 bp indel upstream of the gene and frame-shift mutations within the protein coding region (c.597delC, c.827_828insCC, and c.830_840delGTGTCAGCAGC, where the positions refer to the protein coding sequence for the prairie vole *Avpr1a* annotated in GenBank Ac# AF069304).

A previous study reported that the *Avpr1a* locus was duplicated in the prairie vole but not in the montane vole (*Microtus montanus*) [6]. The low divergence between the duplicated *Avpr1a* loci we observed in this study and the size of the duplicated region (≥105 kb) is therefore consistent with a recent segmental duplication of this region having occurred in the prairie vole lineage. The frameshift mutations in the truncated copy of *Avpr1a* suggests that it is now a pseudogene, which is a common evolutionary fate for newly duplicated genes [38]. Characterization of *Avpr1a* in additional species will be needed to better reconstruct the history of this duplication and the phylogenetic distribution and fate of the duplicated copy of this gene in other voles.

### Conclusions

The ability to study genes and their molecular and genetic correlates with behavior is dependent in part on the availability of genetic and sequence resources. In this study we have generated genomic sequence, the predicted cDNA and protein sequences for 21 behaviorally-relevant genes in the prairie vole, and identified a large number of linked polymorphisms. Combined, these data can be used as a starting platform for future studies focused on characterizing the role of these genes in behavioral phenotypes in the prairie vole, such as genetic association studies, quantification of gene transcript levels and expression patterns, as well as scans for *cis*-regulatory elements. In addition, our results provided novel information as to the genetic diversity within the prairie vole and candidate lineage-specific changes to a number of behaviorally-relevant proteins.

## Supporting Information

File S1 Sequences used in the analyses of the prairie vole proteins.
(DOC)

Table S1 GenBank accession numbers for assembled and annotated prairie vole sequences.
(DOC)

Table S2 Amino acid substitutions specific to the prairie vole lineage.
(DOC)

Table S3 Nonsynonymous variants identified in the behaviorally-relevant prairie vole proteins.
(DOC)
