# Supplementary material for: BAC-Based Sequencing of Behaviorally-Relevant Genes in the Prairie Vole
Source: PLoS One. 2012 Jan 6;7(1):e29345. doi: 10.1371/journal.pone.0029345 (PMC3253076; doi:10.1371/journal.pone.0029345)
Supplement: File S1 — Sequences used in the analyses of the prairie vole proteins. (DOC) [file pone.0029345.s001.doc]

**Supplementary File S1.**

Sequences used in the analyses of the prairie vole proteins.

mo = *Microtus ochrogaster*

mmu = *Mus musculus*

rn = *Rattus norvegicus*

cp = *Cavia porcellus*

oc = *Oryctolagus cuniculus*

>moAR partial, internal gap

MEVQLGPGRVYPRPPSKTYRGAFQNLFQNVREAIQNPGPRHPEA

ASTAPPGACLQQQQQQQQQQQQQQQQQQQQETSPQQRRRQQLGEDGSPQAHIRGPTGY

LALEEEQQPSQQHSASEGHPESGCLPEPGTATAPGKGLLQQPPAPPDEDDSAAPSTLS

LLGPTFPGLSSCSADIKDILSDAGTMQLLQQQQQQQQQQQQQQQQEVISESSGSSVRA

REATGVPSSSKDSYLGGNSTISDSAKELCKAVSVSMGLGVEALEHLSPGEQLRGDCMY

ASLLGGPPAVRPAPCAPLAECKGLPLDEGPGKGTEETAEYSSFKGGYARGLESESLVC

SGSGEAGSSGTLEIPSTLSLYKSGVVDEAAAYQNRDYYNFPLALTGPPHPQPPTHPHA

RIKLENPLDYGSAWAAAAAAQCRYGDLASLHGGSAVGPSTGSPPATASSSWHTLFTTE

EGQLYGPGGRGGSSSPSEAGPVVPYGYTHPPQGLVSQEGDFSASEVWYPTGVVNRVPY

PSSSCVKSEMGPWMENYSGPYGDMRLDSARDHVLPIDYYFPPQKTCLICGDEASGCHY

GALTCGSCKVFFKRAAERKLKKLGNLKLQEEGENSNAGSPTEDPCQKLTVSHVEGYECQPI

FLNVLEAIEPGVVCAGHDNNKPDSFAALLTSLNELGERQLVHVVKWAKALPGFRNLHV

DDQMAVIQYSWMGLMVFAMGWRSFTNVNSRMLYFAPDLVFNEYRMHKSRMYSQCVRMR

QLSQEFGWLQITPQEFLCMKALLLFSIIPVDGLKNQKFFDELRMNYIKELDRIIACKR

KNPTSCSRRFYQLTKLLDSVQPIARELHQFTFDLLIKSHMVSVDFPEMMAEIISVQVP

KILSGKVKPIYFHAQ

>mmuAR gi|7304901|ref|NP_038504.1| androgen receptor [Mus musculus]

MEVQLGLGRVYPRPPSKTYRGAFQNLFQSVREAIQNPGPRHPEAANIAPPGACLQQRQETSPRRRRRQQH

TEDGSPQAHIRGPTGYLALEEEQQPSQQQAASEGHPESSCLPEPGAATAPGKGLPQQPPAPPDQDDSAAP

STLSLLGPTFPGLSSCSADIKDILNEAGTMQLLQQQQQQQQHQQQHQQHQQQQEVISEGSSARAREATGA

PSSSKDSYLGGNSTISDSAKELCKAVSVSMGLGVEALEHLSPGEQLRGDCMYASLLGGPPAVRPTPCAPL

PECKGLPLDEGPGKSTEETAEYSSFKGGYAKGLEGESLGCSGSSEAGSSGTLEIPSSLSLYKSGALDEAA

AYQNRDYYNFPLALSGPPHPPPPTHPHARIKLENPLDYGSAWAAAAAQCRYGDLGSLHGGSVAGPSTGSP

PATTSSSWHTLFTAEEGQLYGPGGGGGSSSPSDAGPVAPYGYTRPPQGLTSQESDYSASEVWYPGGVVNR

VPYPSPNCVKSEMGPWMENYSGPYGDMRLDSTRDHVLPIDYYFPPQKTCLICGDEASGCHYGALTCGSCK

VFFKRAAEGKQKYLCASRNDCTIDKFRRKNCPSCRLRKCYEAGMTLGARKLKKLGNLKLQEEGENSNAGS

PTEDPSQKMTVSHIEGYECQPIFLNVLEAIEPGVVCAGHDNNQPDSFAALLSSLNELGERQLVHVVKWAK

ALPGFRNLHVDDQMAVIQYSWMGLMVFAMGWRSFTNVNSRMLYFAPDLVFNEYRMHKSRMYSQCVRMRHL

SQEFGWLQITPQEFLCMKALLLFSIIPVDGLKNQKFFDELRMNYIKELDRIIACKRKNPTSCSRRFYQLT

KLLDSVQPIARELHQFTFDLLIKSHMVSVDFPEMMAEIISVQVPKILSGKVKPIYFHTQ

>rnAR gi|202968|gb|AAA40759.1| androgen receptor [Rattus norvegicus]

MEVQLGLGRVYPRPPSKTYRGAFQNLFQSVREAIQNPGPRHPEAASIAPPGACLQQRQETSPRRRRRQQH

PEDGSPQAHIRGTTGYLALEEEQQPSQQQSASEGHPESGCLPEPGAATAPGKGLPQQPPAPPDQDDSAAP

STLSLLGPTFPGLSSCSADIKDILSEAGTMQLLQQQQQQQQQQQQQQQQQQQQQQEVISEGSSSVRAREA

TGAPSSSKDSYLGGNSTISDSAKELCKAVSVSMGLGVEALEHLSPGEQLRGDCMYASLLGGPPAVRPTPC

APLAECKGLSLDEGPGKGTEETAEYSSFKGGYAKGLEGESLGCSGSSEAGSSGTLEIPSSLSLYKSGAVD

EAAAYQNRDYYNFPLALSGPPHPPPPTHPHARIKLENPLDYGSAWAAAAAQCRYGDLASLHGGSVAGPST

GSPPATASSSWHTLFTAEEGQLYGPGGGGGSSSPSDAGPVAPYGYTRPPQGLASQEGDFSASEVWYPGGV

VNRVPYPSPSCVKSEMGPWMENYSGPYGDMRLDSTRDHVLPIDYYFPPQKTCLICGDEASGCHYGALTCG

SCKVFFKRAAEGKQKYLCASRNDCTIDKFRRKNCPSCRLRKCYEAGMTLGARKLKKLGNLKLQEEGENSS

AGSPTEDPSQKMTVSHIEGYECQPIFLNVLEAIEPGVVCAGHDNNQPDSFAALLSSLNELGERQLVHVVK

WAKALPGFRNLHVDDQMAVIQYSWMGLMVFAMGWRSFTNVNSRMLYFAPDLVFNEYRMHKSRMYSQCVRM

RHLSQEFGWLQITPQEFLCMKALLLFSIIPVDGLKNQKFFDELRMNYIKELDRIIACKRKNPTSCSRRFY

QLTKLLDSVQPIARELHQFTFDLLIKSHMVSVDFPEMMAEIISVQVPKILSGKVKPIYFHTQ

>cpAR ENSCPOT00000007709 length=853

MEVQLGLGRVYPRPPSKTFRGAFQNLFQSVREVIQTPGPRHPEADNAIPP

SAHSENGSPQAHIRGPTGYLALEEERQPAPQQSTPEGHPERGCISEPGTS

VPASKGLPQQPPVPPDEDDSAAPSTLSLLGPTFPSLSSCSADLKDILSEA

RMEVVSETGSSGRAREAVGAATSSKDSYLGVNSTISDSAKELCKAVSVSM

GLGVEALDHLSPGEQLRGDCMYAPLLGGPPPVRPTHCASLVECKGSLLND

SPDKGTEETVEYSSFKGGYSKSLEGETLGCSGSGDAGSSGTLELPPTLSL

YKSGALDEAASYQNRDYYNFPLPPPPPPHPNARIKLENPLDYSSAWSAAA

AQCRYGDLASLHGGGAAGPSSGSPSATASSSWHTLFTTEEGQLYGPCGGG

GGGSTSETGAVAPYGYSRPPQGLAGQEGDFPAPDMWYPGGMAGRVPYPSP

SCVKSEIGPWMESYSGPYGDVRLENARDHVLPIDYYFPPQKTCLICGDEA

SGCHYGALTCGSCKVFFKRAAEGKQKYLCASRNDCTIDKFRRKNCPSCRL

RKCYEAGMTLGARKLKKLGNLKLQEEGEASSSSSPTEEPCQKMTVSHMEG

YECQPIFLNVLEAIEPGVVCAGHDNNQPDSFAALLSSLNELGERQLVHVV

KWAKALPGFRNLHVDDQMAVIQYSWMGLMVFAMGWRSFTNVNSRMLYFAP

DLVFNEYRMHKSRMYSQCVRMRHLSQEFGWLQITPQEFLCMKALLLFSII

PVDGLKNQKFFDELRMNYIKELDRIIACKRKNPTSCSRRFYQLTKLLDSV

QPIARELHQFTFDLLIKSHMVSVDFPEMMAEIISVQVPKILSGKVKPIYF

HTQ

>ocAR ENSOCUT00000003141 length=843

MEVQLGLGRVYPRPPSKTYRGAFQNLFQNVREVIQNPSPRHPEAASAAPP

GARSEDGSPQAQIRGPTGYLALEERQQPSHQQPAPEGRPENSCVPEPGAA

VAGGKGLPQQPAAPPDEDDSAAPSTLSLLGPTFPGLNSCSADLKDILSEG

AREAAGASTSSKDSYLGSTSVISDSAKELCKAVSVSMGLGVEALEHLSSG

EQLRGDCMYAPLLGGPPVVRPTPCLPLVECKGSLLDDGPGKGTEETAEYT

PFKGGYNKGLEAESLGCSGSGEAGSSGTLELPSTLSLYKSGTLDEAAAYQ

TRDYYNFPLALAGQPPPPHPHARIKLENPLDYGSAWAAAAAQCRYGDLAS

LHGGGAAGPGSGSPSTAASSSWHTLFTTEEGQLYGPCGGGGGSGPGEAGA

VAPYGYTRPPQGLTGQEGDFPAPEVWYPGGVVSRVPYPNPSCVKSEMGPW

MESYSGPYGDMRLETARDHVLPIDYYFPPQKTCLICGDEASGCHYGALTC

GSCKVFFKRAAEGKQKYLCASRNDCTIDKFRRKNCPSCRLRKCYEAGMTL

GARKLKKLGNLKLQEEGESSSASSPTEDTTQKLTVSHMEGYECQPIFLNV

LEAIEPGVVCAGHDNNQPDSFAALLSSLNELGERQLVHVVKWAKALPGFR

NLHVDDQMAVIQYSWMGLMVFAMGWRSFTNVNSRMLYFAPDLVFNEYRMH

KSRMYSQCVRMRHLSQEFGWLQITPQEFLCMKALLLFSIIPVDGLKNQKF

FDELRMNYIKELDRIIACKRKNPTSCSRRFYQLTKLLDSVQPIARELHQF

TFDLLIKSHMVSVDFPEMMAEIISVQVPKILSGKVKPIYFHTQ

>moAVP_1

MLARMLNSTLSACFLSLLAFTSACYFQNCPRGGKRAMPDMELRQ

CLPCGPGDQGRCFGPNICCADELGCFVGTAEALRCQEENYLPSPCQSGQKPCGSGGRC

AAAGICCSDESCVTDPECREDFLRLTRAREPNNGTQLDGPARALLLRLLQMAGTRESV

DSAKPRVY

>moAVP_2

MLARMLNSTLSACFLSLLAFTSACYFQNCPRGGKRAMPDMELRQ

CLPCGPGDQGRCFGPNICCADELGCFVGTAEALRCQEENYLPSPCQSGQKPCGSGGRC

AAAGICCSDESCVSDPECREDFLRLTSAREPNNGTQLDGPARALLLRLLQMAGTRESV

DSAKPRVY

>mmuAVP NP_033862 length=168

MLARMLNTTLSACFLSLLAFSSACYFQNCPRGGKRAISDMELRQCLPCGP

GGKGRCFGPSICCADELGCFVGTAEALRCQEENYLPSPCQSGQKPCGSGG

RCAAVGICCSDESCVAEPECHDGFFRLTRAREPSNATQLDGPARALLLRL

VQLAGTRESVDSAKPRVY

>rnAVP NP_058688 length=168

MLAMMLNTTLSACFLSLLALTSACYFQNCPRGGKRATSDMELRQCLPCGP

GGKGRCFGPSICCADELGCFLGTAEALRCQEENYLPSPCQSGQKPCGSGG

RCAAAGICCSDESCVAEPECREGFFRLTRAREQSNATQLDGPARELLLRL

VQLAGTQESVDSAKPRVY

>cpAVP ENSCPOT00000027329 length=160

MPDAMLPACLLGLLAFTSACYFQNCPRGGKRALSDTELRQCGPGGQGRCF

GPSICCADALGCFVGTAEALRCQEENYLPSPCQSGQKPCGSGGRCAANGV

CCNDESCVIEPECREEFHRPVRAGDRSNVTQLDGPAGALLLRLMQLATKP

RPQNPEPATW

>ocAVP ENSOCUT00000001770 length=168

VCARMPDTMLPVCFLGLLACASACYFQNCPRGGKRAMSDLELRQCLPCGP

AGKGRCFGPSICCADELGCFVGTAEALRCQEENFLPSPCQSGQKPCGSGG

RCAAAGVCCNDESCVTDPECREAFPRRARASDRSNATQLDAPAGALLLRL

VQLAGAPEPAEPAKPGGY

>moAVPR1A

MSFPRGSYDPAASNSSPWWPLSAEDANSSWEAAGHQKGSDPSGDVRNEEL

AKLEIAVLAVIFVVAVLGNSSVLLALHRTPRKTSRMHLFIRHLSLADLAV

AFFQVLPQLCWDITYRFRGPDWLCRVVKHLQVFAMFASAYMLVVMTADRY

IAVCHPLKTLQQPTRRSRLMIAASWVLSFLLSTPQYFIFSMIEIEVNNGT

KTQDCWATFIQPWGTRAYVTWMTSGVFVVPVVILGTCYGFICYHIWRNVR

GKTASRQSKGSGEDVAPFHKGLLVTPCVSSVKTISRAKIRTVKMTFVIVT

AYILCWAPFFIVQMWSVWDDNFIWTDSENPSITITALLASLNSCCNPWIY

MFFSGHLLQDCVQSFPCCQRMVQKFTKDDSDNMSRRQTSYSNNRSPTNST

GTWKDSPKSSRSIRFIPVST

>mmuAVRR1A ENSMUST00000020323 length=423

MSFPRGSHDLPAGNSSPWWPLTTEGANSSREAAGLGEGGSPPGDVRNEEL

AKLEVTVLAVIFVVAVLGNSSVLLALHRTPRKTSRMHLFIRHLSLADLAV

AFFQVLPQLCWDITYRFRGPDWLCRVVKHLQVFAMFASSYMLVVMTADRY

IAVCHPLKTLQQPARRSRLMIAASWGLSFVLSIPQYFIFSVIEFEVNNGT

KAQDCWATFIPPWGTRAYVTWMTSGVFVVPVIILGTCYGFICYHIWRNVR

GKTASRQSKGGKGSGEAAGPFHKGLLVTPCVSSVKSISRAKIRTVKMTFV

IVSAYILCWTPFFIVQMWSVWDTNFVWTDSENPSTTITALLASLNSCCNP

WIYMFFSGHLLQDCVQSFPCCQSIAQKFAKDDSDSMSRRQTSYSNNRSPT

NSTGTWKDSPKSSKSIRFIPVST

>rnAVPR1A NP_444178 length=424

MSFPRGSQDRSVGNSSPWWPLTTEGSNGSQEAARLGEGDSPLGDVRNEEL

AKLEIAVLAVIFVVAVLGNSSVLLALHRTPRKTSRMHLFIRHLSLADLAV

AFFQVLPQLCWDITYRFRGPDWLCRVVKHLQVFAMFASAYMLVVMTADRY

IAVCHPLKTLQQPARRSRLMIATSWVLSFILSTPQYFIFSVIEIEVNNGT

KTQDCWATFIQPWGTRAYVTWMTSGVFVAPVVVLGTCYGFICYHIWRNIR

GKTASSRHSKGDKGSGEAVGPFHKGLLVTPCVSSVKSISRAKIRTVKMTF

VIVSAYILCWAPFFIVQMWSVWDENFIWTDSENPSITITALLASLNSCCN

PWIYMFFSGHLLQDCVQSFPCCHSMAQKFAKDDSDSMSRRQTSYSNNRSP

TNSTGMWKDSPKSSKSIRFIPVST

>cpAVPR1A ENSCPOT00000012202 peptide: ENSCPOP00000010869 pep:NOVEL_protein_coding

MRFSGNAVADPAGNSSAWWALTADGANSSREADVPREGDCQPRDVRNEEL

AKVEIAVLAVTFVVAVLGNSSVLLALHRTPRKTSRMHLFIRHLSLADLAV

AFFQVLPQLCWDVTYRFRGPDWLCRVVKHLQVFAMFASAYMLVVMTADRY

IAVCHPLKTLQQPARRSRLMIAASWVLSFVLSTPQYFIFSMIEVDNVTKA

QDCWATFIQPWGSRAYVTWMTSCVFVAPVVILGTCYGFICYHIWRNVRGK

TASRLGEGAAGSGGAFHKGLLIAPFVSSVKTISRAKIRTVKMTFVIVTVY

ILCWAPFIIVQMWSVWDEKFVWTDSENPTTTITALLASLNSCCNPWIYMF

FSGHLLQDCVKSFPCCQSMKQKINKEDTDSMSRRQTSYFNNRSPTNSTGT

WKDSPKSSKSSKFIPVST

>ocAVPR1A gi|291389479|ref|XP_002711283.1| PREDICTED: arginine vasopressin receptor 1A [Oryctolagus cuniculus]

MRFSGSSDVEPAGNFSPWWPLRAGGANGSLGAGEPPGEGDEPQGDVRNEELAKLEIAVLAVTFAVAVLGN

ISVLLALHRTPRKTSRMHLFIRHLSLADLAVAFFQVLPQLCWDITYRFRGPDWLCRVVKHLQVFAMFASA

YMLVVMTADRYIAVCHPLKTLQQPARRSRLMIAASWVLSFVLSTPQYFIFSMIEVNNVTKAQDCWATFIQ

PWGSRAYVTWMTSGIFVAPVVILGTCYGFICYHIWSNVRGKTASRQSKGSEGAAGAFHKGLLLAPCVSSV

KTISRAKIRTVKMTFVIVTAYIVCWAPFFIIQMWSVWDENFNWTDSENPSITITALLASLNSCCNPWIYM

FFSGHLLQDCLQSFLCCQNIKQNFNKEETDSMSRRQTSYSNNRSPTNSTGTWKDSPKSSKSIKVLPAST

>moBDNF

MFHQVRRVMTILFLTMVISYLGCMKAAPMKEANVHGQGSLAYPG

VRTHGTLESVNGPRAGSRGLTTSLADTFEHVIEELLDEDQKVRPNGVNHKDADLYTSR

VMLSSQVPLEPPLLFLLEEYKNYLDAANMSMRVRRHSDPARRGELSVCDSISEWVTAA

DKKTAVDMSGGTVTVLEKVPVSKGQLKQYFYETKCNPMGYTKEGCRGIDKKHWNSQCR

TTQSYVRALTMDSKKRIGWRFIRIDTSCVCTLTIKRGR

>mmuBDNF NP_031566 length=257

MFHQVRRVMTILFLTMVISYFGCMKAAPMKEVNVHGQGNLAYPGVRTHGT

LESVNGPRAGSRGLTTTSLADTFEHVIEELLDEDQKVRPNEENHKDADLY

TSRVMLSSQVPLEPPLLFLLEEYKNYLDAANMSMRVRRHSDPARRGELSV

CDSISEWVTAADKKTAVDMSGGTVTVLEKVPVSKGQLKQYFYETKCNPMG

YTKEGCRGIDKRHWNSQCRTTQSYVRALTMDSKKRIGWRFIRIDTSCVCT

LTIKRGR

>rnBDNF NP_036645 length=249

MTILFLTMVISYFGCMKAAPMKEANVHGQGNLAYPAVRTHGTLESVNGPR

AGSRGLTTTSLADTFEHVIEELLDEDQKVRPNEENHKDADLYTSRVMLSS

QVPLEPPLLFLLEEYKNYLDAANMSMRVRRHSDPARRGELSVCDSISEWV

TAADKKTAVDMSGGTVTVLEKVPVSKGQLKQYFYETKCNPMGYTKEGCRG

IDKRHWNSQCRTTQSYVRALTMDSKKRIGWRFIRIDTSCVCTLTIKRGR

>cpBDNF ENSCPOG00000009920:ENSCPOT00000010009 peptide: ENSCPOP00000017078 pep:KNOWN_protein_coding

MTILFLTMVISYFGCMKAAPMKEASVRGPGSLAYPGVRTHGALESATGPKVGARGLTSSS

SSSSSSLADTFEHVIEELLVEDQKARPHEESAKDADLYTSRVMLSSQVPLEPPLLFLLEE

YKNYLDAANMSMRVRRHSDPARRGELSVCDSVSEWVTAADKKTAVDMSGGTVTVLEKVPV

SKGQLKQYFYETKCNPMGYTKEGCRGIDKRHWNSQCRTTQSYVRALTMDSKKRIGWRFIR

IDTSCVCTLTIKRGR

>ocBDNF ENSOCUT00000006729 length=255

QFHQVRRVMTILFLTMVISYFGCMKAAPMKEANVRGQGSLAYPGMRTHGT

LESVNGPKTGSRGLTSLADTFEHVIEELLDEDQKVRPNEENNKDADLYTS

RVMLSSQVPLEPPLLFLLEEYKNYLDAANMSMRVRRHSDPARRGELSVCD

SISEWVTAADKKTAVDMSGGTVTVLEKVPVSKGQLKQYFYETKCNPMGYT

KEGCRGIDKRHWNSQCRTTQSYVRALTMDSKKRIGWRFIRIDTSCVCTLT

IKRGR

>moCRH

MRLRLLVSVGILLMALSPSPPCRALLSRGPVPGAPRSPQPLNFLQPEQPQ

QPQPVLIRMGEEYFLRLGNLNRSPAARLSPDSSPLFAGRGSRPSHDQAAA

NFFRVLLQQLQTPQRSLDSRSGPAERGAEDTLGGHQGALERERRSEEPPI

SLDLTFHLLREVLEMARAEQLAQQAHSNRKLMEIIGK

>mmuCRH ENSMUST00000061294 length=187

MRLRLLVSAGMLLVALSSCLPCRALLSRGSVPRAPRAPQPLNFLQPEQPQ

QPQPVLIRMGEEYFLRLGNLNRSPAARLSPNSTPLTAGRGSRPSHDQAAA

NFFRVLLQQLQMPQRSLDSRAEPAERGAEDALGGHQGALERERRSEEPPI

SLDLTFHLLREVLEMARAEQLAQQAHSNRKLMEIIGK

>rnCRH ENSRNOT00000016953 length=187

MRLRLLVSAGMLLVALSPCLPCRALLSRGSVSGAPRAPQPLNFLQPEQPQ

QPQPILIRMGEEYFLRLGNLNRSPAARLSPNSTPLTAGRGSRPSHDQAAA

NFFRVLLQQLQMPQRPLDSSTELAERGAEDALGGHQGALERERRSEEPPI

SLDLTFHLLREVLEMARAEQLAQQAHSNRKLMEIIGK

>cpCRH ENSCPOT00000001659 length=193

MRLPLLVSAGVLLVALLPGPPCRALLSRGPVPGAPQVPRSPQPLDFFQPP

PQPEQLRPPQSRPVLLRVGEEYFLRLGSLSRSPAARFAPGASPLAGGSRR

SPDLAAVNFFRALQQPQTPLRSLDSPADPAQRRGGSALGGHQEALERERR

SEEPPISLDLTFHLLREVLEMARAEQLAQQAHNNRKLMEIIGK

>ocCRH gi|291388060|ref|XP_002710492.1| PREDICTED: corticotropin releasing hormone [Oryctolagus cuniculus]

MRLPLLVSAGVLLVALLPCPPCRALLRRGPVPGVPQAPQPLDFFQPQPPPSQPQQPQPRPILLRMGEEYF

LRLGNLGRSPVAALSPASSPLAGAGSGGRPLPDQAAANFFRVLLQQLPLSGRSLDSRAGPSERGAESALG

GHQEAPERERRSEEPPISLDLTFHLLREVLEMARAEQLAQQAHSNRKLMEIIGK

>moCRHR1_1 partial

MGRRPQLRLVK

LHCNASVDLIGTCWPRSPAGQLVVRPCPAFFYGVRYNTTNNGYR

ECLANGSWAARVNYSECQEILNEEKKSKVHYHIAVIINYLGHCISLVALLVAFVLFLW

LRSIRCLRNIIHWNLISAFILRNATWFVVQLTMNPEVHQSNVGWCRLVTAAYNYFHVT

NFFWMFGEGCYLHTAIVLTYSTDRLRKWMFVCIGWGVPFPIIVAWAIGKLYYDNEKCW

FGKRPGVYTDYIYQGPMILVLLINFIFLFNIVRILMTKLRASTTSETIQYRKAVKATL

VLLPLLGITYMLFFVNPGEDEVSRIVFIYFNSFLESFQGFFVSVFYCFLNSEVRSAIR

KRWHRWQDKHSIRARVARAMSIPTSPTRVSFHSIKQSSAV

>moCRHR1_2 partial

KKSKVHYHIAVIINYLGHCISLVALLAAFVLFLRLRSIRCLRNI

IHWNLISAFILRNATWFVVQLTMNPEVHQSNVGWCRLVTAAYNYFHVTNFFWMFGEGC

YLHTAIVLTYSTDRLRKWMFICIGWGVPFPIIVAWAIGKLYYDNEKCWFGKRPGVYTD

YIYQGPMILVLLINFIFLFNIVRILMTKLRASTTSETIQYRKAVKATLVLLPLLGITY

MLFFVNPGEDEVSRIVFIYFNSFLESFQGFFVSVFYCFLNSEVRSAIRKRWHRWQDKH

SIRARVARAMSIPTSPTRVSFHSIKQSSAV

>mmuCRHR1 NP_031788 length=415

MGQRPQLRLVKALLLLGLNPVSTSLQDQQCESLSLASNVSGLQCNASVDL

IGTCWPRSPAGQLVVRPCPAFFYGVRYNTTNNGYRECLANGSWAARVNYS

ECQEILNEEKKSKVHYHIAVIINYLGHCISLVALLVAFVLFLRLRSIRCL

RNIIHWNLISAFILRNATWFVVQLTVSPEVHQSNVAWCRLVTAAYNYFHV

TNFFWMFGEGCYLHTAIVLTYSTDRLRKWMFVCIGWGVPFPIIVAWAIGK

LYYDNEKCWFGKRPGVYTDYIYQGPMILVLLINFIFLFNIVRILMTKLRA

STTSETIQYRKAVKATLVLLPLLGITYMLFFVNPGEDEVSRVVFIYFNSF

LESFQGFFVSVFYCFLNSEVRSAIRKRWRRWQDKHSIRARVARAMSIPTS

PTRVSFHSIKQSTAV

>rnCRHR1 NP_112261 length=415

MGRRPQLRLVKALLLLGLNPVSTSLQDQRCENLSLTSNVSGLQCNASVDL

IGTCWPRSPAGQLVVRPCPAFFYGVRYNTTNNGYRECLANGSWAARVNYS

ECQEILNEEKKSKVHYHVAVIINYLGHCISLVALLVAFVLFLRLRSIRCL

RNIIHWNLISAFILRNATWFVVQLTVSPEVHQSNVAWCRLVTAAYNYFHV

TNFFWMFGEGCYLHTAIVLTYSTDRLRKWMFVCIGWGVPFPIIVAWAIGK

LHYDNEKCWFGKRPGVYTDYIYQGPMILVLLINFIFLFNIVRILMTKLRA

STTSETIQYRKAVKATLVLLPLLGITYMLFFVNPGEDEVSRVVFIYFNSF

LESFQGFFVSVFYCFLNSEVRSAIRKRWRRWQDKHSIRARVARAMSIPTS

PTRVSFHSIKQSTAV

>cpCRHR1 ENSCPOT00000005594 length=384

SSSPAVSTPGLQCNASVDLIGTCWPRSPAGQLVVRPCPAFFYGVRYNTTN

NGYRECLANGSWAARVNYSECQEILNEEKKSKVHYQIAVIINYLGHCISL

VALLVAFILFLRLRSIRCLRNIIHWNLISAFILRNATWFVVQRTMSPEVH

QSNVGWCRLVTAAYNYFHVTNFFWMFGEGCYLHTAIVLTYSTDRLRKWMF

ICIGWGVPFPIIVAWAIGKLYYDNEKCWFGKRPGVYTDYIYQGPMILVLL

INFIFLFNIVRILMTKLRASTTSETIQYRKAVKATLVLLPLLGITYMLFF

VNPGEDEVSRVVFIYFNSLLESFQGFFVSVFYCFLNSEVRSAIRKRWHRW

QDKHSIRARVARAMSIPTSPTRVSFHSIKQSTAV

>ocCRHR1 NP_001156587 length=415

MGRRPQLPLVKALLLLGLNPVSASLQDQHCESLSPASNVSGLQCNASVDL

IGTCWPRSPAGQLVVRPCPAFFYGVRYNTTNNGYRECLANGSWAARVNYS

ACQEILNEEKKSKVHYHVAVIINYLGHCVSLVALLVAFVLFLRLRSIRCL

RNIIHWNLISAFILRNATWFVVQLTMSPEVHQSNVGWCRLVTAAYNYFHV

TNFFWMFGEGCYLHTAIVLTYSTDRLRKWMFVCIGWGVPFPIIVAWAIGK

LYYDNEKCWFGKRPGVYTDYIYQGPMILVLLINFIFLFNIVRILMTKLRA

STTSETIQYRKAVKATLVLLPLLGITYMLFFVHPGEDEVSRVVFIYFNSF

LESFQGFFVSVFYCFLNSEVRSAIRKRWHRWQDKHSIRARVARAMSIPTS

PTRVSFHSIKQSTAV

>moCRHR2

MGAPGHPPSAQLLCLLCLLLLLQVVRPGQVLQDQPLWTLLEQHC

HRTTKLINLSGSYSYCNTTLDQIGTCWPQSTPGALVERPCPEYFNGIKYNTTRNAYRE

CLENGTWASRINYSHCEPILDDKRKYDLHYRIALIVNYLGHCVSVVALVAAFLLFLFL

RSIRCLRNVIHWNLITTFILRNVTWFLLQLIDHEVHEGNEVWCRCITTIFNYFVVTNF

FWMFVEGCYLHTAIVMTYSTEHLRKWLFLFIGWCIPCPIIIAWALGKLYYENEQCWFG

KEPGDLVDYIYQGPIILVLLINFIFLFNIVRILMTKLRASTTSETIQYRKAVKATLVL

LPLLGITYMLFFVNPGEDDLSQIVFIYFNSFLQSFQGFFVSVFYCFFNGEVRSALRKR

WHRWQDHHSLRVPVARAMSIPTSPTRISFHSIKQTAAV

>mmuCRHR2 ENSMUST00000003568 length=430

MGTPGSLPSAQLLLCLFSLLPVLQVAQPGQAPQDQPLWTLLEQYCHRTTT

GNFSGPYTYCNTTLDQIGTCWPQSAPGALVERPCPEYFNGIKYNTTRNAY

RECLENGTWASRVNYSHCEPILDDKRKYDLHYRIALIVNYLGHCVSVVAL

VAAFLLFLVLRSIRCLRNVIHWNLITTFILRNIAWFLLQLIDHEVHEGNE

VWCRCITTIFNYFVVTNFFWMFVEGCYLHTAIVMTYSTEHLRKWLFLFIG

WCIPCPIIIAWAVGKLYYENEQCWFGKEAGDLVDYIYQGPVMLVLLINFV

FLFNIVRILMTKLRASTTSETIQYRKAVKATLVLLPLLGITYMLFFVNPG

EDDLSQIVFIYFNSFLQSFQGFFVSVFYCFFNGEVRAALRKRWHRWQDHH

ALRVPVARAMSIPTSPTRISFHSIKQTAAV

>rnCRHR2 ENSRNOT00000014925 length=431

MGTPGSLPSAQLLLCLYSLLPLLQVAQPGQALQDQPLWTLLEQYCHRTTT

RNFSGPYSYCNTTLDQIGTCWPQSAPGALVERPCPEYFNGIKYNTTRNAY

RECLENGTWASRVNYSHCEPILDDKQRKYDLHYRIALIINYLGHCVSVVA

LVAAFLLFLVLRSIRCLRNVIHWNLITTFILRNITWFLLQLIDHEVHEGN

EVWCRCVTTIFNYFVVTNFFWMFVEGCYLHTAIVMTYSTEHLRKWLFLFI

GWCIPCPIIVAWAVGKLYYENEQCWFGKEPGDLVDYIYQGPIILVLLINF

VFLFNIVRILMTKLRASTTSETIQYRKAVKATLVLLPLLGITYMLFFVNP

GEDDLSQIVFIYFNSFLQSFQGFFVSVFYCFFNGEVRSALRKRWHRWQDH

HALRVPVARAMSIPTSPTRISFHSIKQTAAV

>cpCRHR2 ENSCPOG00000020502:ENSCPOT00000026865 peptide: ENSCPOP00000018868 pep:NOVEL_protein_coding

MDAALFHSLLEANCSLVLAEELLLDGWGPPLDPEGPYSYCNTTLDQIGTCWPQSVAGALV

ERPCPEYFNGIKYNTTRNAYRECLENGTWASKINYSQCEPILDDKQRKYDLHYRIALVVN

YLGHCISMVALVAAFLLFLALRSIRCLRNVIHWNLVTTFILRNVTWFLLQLIDHEVHESN

EVWCRCITTVFNYFVVTNFFWMFVEGCYLHTAIVMTYSTERLRKWIFLFIGWCVPCPIII

AWAIGKLYYEDKQCWFGKEPGDLMDYIYQGPIIFVLLINFVFLFNIVRILMTKLRASTTS

ETIQYRKAVKATLVLLPLLGITYMLFFVNPGEDELSQIVFIYFNSFLQSFQGFFVSVFYC

FFNGEVRSAVRKRWHRWQDHYSLRVPVARAMSIPTSPTRISFHSIKQTAAV

>ocCRHR2 gi|291394615|ref|XP_002713782.1| PREDICTED: corticotropin releasing factor type 2A receptor-like [Oryctolagus cuniculus]

MDAALLHSLLEANCSLALAEELLLDGWGQPLDPEGPDSYCNTTLDQIGTCWPRSAAGALVERPCPEYFNG

VKYNTTRNAYRECLENGTWALRINYSQCEPIFDDKRKYDLHYRIALVVNYLGHCVSMAALVAAFLLFLAL

RSIRCLRNVIHWNLITTFIRRNVAWFLLQLIDHEVHESNEVWCRCITATFNYFVVTNFFWMFVEGCYLHT

AIAMTYSTERLRKWLFLFIGWCIPCPIIIAWAVGKLYYENEQCWFGKEPGDLVDYIYQGPIILVLLINFV

FLFNIVRILMTKLRASTTSETMQYRKAVKATLVLLPLLGITYMLFFINPGEDDLAQIVFIYFNSFLQSFQ

GFFVSVFYCFFNGEVRSALRKRWHRWQDHHSLRVPVARGMSIPTSPTRISFHSIKHTAAV

>moDRD1A_1

MALNTSTMDGAGLAVERDFSFRILTACFLSLLILSTLLGNTLVC

AAVIRFRHLRSKVTNFFVISLAVSDLLVAVLVMPWKAVAEIAGFWPFGSFCNIWVAFD

IMCSTASILNLCVISVDRYWAISSPFQYERKMTPKAAFILISVAWTLSVLISFIPVQL

SWHKAKPTWPLDDNATFLEDTEDANCDTRLSRTYAISSSLISFYIPVAIMIVTYTSIY

RIAQKQIRRISALERAAVHAKNCQTTTGNGNPVECSQSESSFKMSFKRETKVLKTLSV

IMGVFVCCWLPFFISNCMVPFCGSEETQPFCIDSITFDVFVWFGWANSSLNPIwIYAFN

ADFQKAFSTLLGCYRLCPTTNNAIETVSINNNGAVFSSHHEPRGSISKDCNLVYLIPH

AVGSSEDLKKEEAGGIAKPLEKLSPALSVILDYDTDVSLEKIQPVTHSGQHPT

>mmuDRD1A ENSMUST00000021932 length=446

MAPNTSTMDETGLPVERDFSFRILTACFLSLLILSTLLGNTLVCAAVIRF

RHLRSKVTNFFVISLAVSDLLVAVLVMPWKAVAEIAGFWPFGSFCNIWVA

FDIMCSTASILNLCVISVDRYWAISSPFQYERKMTPKAAFILISVAWTLS

VLISFIPVQLSWHKAKPTWPLDGNFTSLEDAEDDNCDTRLSRTYAISSSL

ISFYIPVAIMIVTYTSIYRIAQKQIRRISALERAAVHAKNCQTTTGNGNP

VECSQSESSFKMSFKRETKVLKTLSVIMGVFVCCWLPFFISNCMVPFCGS

EETQPFCIDSITFDVFVWFGWANSSLNPIIYAFNADFQKAFSTLLGCYRL

CPTTNNAIETVSINNNGAVMFSSHHEPRGSISKDCNLVYLIPHAVGSSED

LKREEAGGIPKPLEKLSPALSVILDYDTDVSLEKIQPVTHSGQHST

>rnDRD1A ENSRNOT00000030893 length=446

MAPNTSTMDEAGLPAERDFSFRILTACFLSLLILSTLLGNTLVCAAVIRF

RHLRSKVTNFFVISLAVSDLLVAVLVMPWKAVAEIAGFWPFGSFCNIWVA

FDIMCSTASILNLCVISVDRYWAISSPFQYERKMTPKAAFILISVAWTLS

VLISFIPVQLSWHKAKPTWPLDGNFTSLEDTEDDNCDTRLSRTYAISSSL

ISFYIPVAIMIVTYTSIYRIAQKQIRRISALERAAVHAKNCQTTAGNGNP

VECAQSESSFKMSFKRETKVLKTLSVIMGVFVCCWLPFFISNCMVPFCGS

EETQPFCIDSITFDVFVWFGWANSSLNPIIYAFNADFQKAFSTLLGCYRL

CPTTNNAIETVSINNNGAVVFSSHHEPRGSISKDCNLVYLIPHAVGSSED

LKKEEAGGIAKPLEKLSPALSVILDYDTDVSLEKIQPVTHSGQHST

>cpDRD1A ENSCPOT00000009587 length=445

KMTLNTSSMDETGLVMERDFSFRILTACFLSLLILSTLLGNTLVCAAVIR

FRHLRSKVTNFFVISLAVSDLLVAVLVMPWKAVAEIAGFWPFGSFCNIWV

AFDIMCSTASILNLCVISVDRYWAISSPFQYERKMTPRAAFILISIAWTL

SVLISFIPVQLSWHKAKPMSPSDGNATFFEEAPDNCDTRLSRTYAISSSL

ISFYIPVAIMIVTYTSIYRIAQKQIRRISALERAAVHAKNCQTTTGNGNV

ECSQSESSFKMSFKRETKVLKTLSVIMGVFVCCWLPFFISNCMVPFCESG

ETQPFCIDSITFDVFVWFGWANSSLNPIIYAFNADFQKAFSTLLGCYRLC

PTTNNAIETVSINNNGAVVFSSHHEPRGSISKECNLVYLIPHAVGSSEDL

KKEEAGGIAKPLEKLSPALSVILDYDTDVSLEKIQPITHNGQHPT

>ocDRD1A gi|291387850|ref|XP_002710254.1| PREDICTED: dopamine receptor D1 [Oryctolagus cuniculus]

MRTLNTSAMDRAGLLVERAFSFRILTACFLSLLILSTLLGNTLVCAAVIRFRHLRSKVTNFFVISLAVSD

LLVAVLVMPWKAVAEIAGFWPFGSFCNIWVAFDIMCSTASILNLCVISVDRYWAISSPFRYERKMTPKAA

FILIGVAWTLSVLISFIPVQLSWHKAKPTSPPDGNATSLDETVDNCDSSLSRTYAISSSLVSFYIPVAIM

IVTYTRIYRIAQKQIRRISALERAAVHAKNCQTTTTTGNGNPVECSQPESSFKMSFKRETKVLKTLSVIM

GVFVCCWLPFFVLNCMVPFCGSGETQSFCIDPITFDVFMWFGWANSSLNPIIYAFNADFRKAFSILLGCY

RLCPTANNAIETVSINNNGAVVFSSHHEPRGSISKECNMVYLIPHAVGSSEDLRKEEAGAIAKPLEKLSP

ALSVILDYDTDVSLEKIQPVTQNGQHPA

>moDRD2_1

MDPLNLSWYDDDLERQNWSRPFNGSEGKADRPHYNYYAMLLTLL

IFIIVFGNVLVCMAVSREKALQTTTNYLIVSLAVADLLVATLVMPWVVYLEVVGEWKF

SRIHCDIFVTLDVMMCTASILNLCAISIDRYTAVAMPMLYNTRYSSKRRVTVMIAIVW

VLSFTISCPLLFGLNNTDQNECIIANPAFVVYSSIVSFYVPFIVTLLVYIKIYIVLRK

RRKRVNTKRSSRAFRANLKTPLKGNCTHPEDMKLCTVIMKSNGSFPVNRRRMDAARRA

QELEMEMLSSTSPPERTRYSPIPPSHHQLTLPDPSHHGLHSNPDSPAKPEKNGHARII

NPRIAKIFEIQTMPNGKTRTSLKSMSRRKLSQQKEKKATQMLAIVLGVFIICWLPFFI

THILNIHCDCNIPPVLYSAFTWLGYVNSAVNPIIYTTFNIEFRKAFMKILHC

>moDRD2_2

MDPLNLSWYDDDLERQNWSRPFNGSEGKVDRPHYNYYAMLLTLL

IFIIVFGNVLVCMAVSREKALQTTTNYLIVSLAVADLLVATLVMPWVVYLEVVGEWKF

SRIHCDIFVTLDVMMCTASILNLCAISIDRYTAVAMPMLYNTRYSSKRRVTVMIAIVW

VLSFTISCPLLFGLNNTDQNECIIANPAFVVYSSIVSFYVPFIVTLLVYIKIYIVLRK

RRKRVNTKRSSRAFRANLKTPLKGNCTHPEDMKLCTVIMKSNGSFPVNRRRMDAARRA

QELEMEMLSSTSPPERTRYSPIPPSHHQLTLPDPSHHGLHSNPDSPAKPEKNGHARII

NPRIAKIFEIQTMPNGKTRTSLKSMSRRKLSQQKEKKATQMLAIVLGVFIICWLPFFI

THILNIHCDCNIPPVLYSAFTWLGYVNSAVNPIIYTTFNIEFRKAFMKILHC

>mmuDRD2 gi|148747212|ref|NP_034207.2| D(2) dopamine receptor [Mus musculus]

MDPLNLSWYDDDLERQNWSRPFNGSEGKPDRPHYNYYAMLLTLLIFIIVFGNVLVCMAVSREKALQTTTN

YLIVSLAVADLLVATLVMPWVVYLEVVGEWKFSRIHCDIFVTLDVMMCTASILNLCAISIDRYTAVAMPM

LYNTRYSSKRRVTVMIAIVWVLSFTISCPLLFGLNNTDQNECIIANPAFVVYSSIVSFYVPFIVTLLVYI

KIYIVLRKRRKRVNTKRSSRAFRANLKTPLKGNCTHPEDMKLCTVIMKSNGSFPVNRRRMDAARRAQELE

MEMLSSTSPPERTRYSPIPPSHHQLTLPDPSHHGLHSNPDSPAKPEKNGHAKIVNPRIAKFFEIQTMPNG

KTRTSLKTMSRRKLSQQKEKKATQMLAIVLGVFIICWLPFFITHILNIHCDCNIPPVLYSAFTWLGYVNS

AVNPIIYTTFNIEFRKAFMKILHC

>rnDRD2 NP_036679 length=444

MDPLNLSWYDDDLERQNWSRPFNGSEGKADRPHYNYYAMLLTLLIFIIVF

GNVLVCMAVSREKALQTTTNYLIVSLAVADLLVATLVMPWVVYLEVVGEW

KFSRIHCDIFVTLDVMMCTASILNLCAISIDRYTAVAMPMLYNTRYSSKR

RVTVMIAIVWVLSFTISCPLLFGLNNTDQNECIIANPAFVVYSSIVSFYV

PFIVTLLVYIKIYIVLRKRRKRVNTKRSSRAFRANLKTPLKGNCTHPEDM

KLCTVIMKSNGSFPVNRRRMDAARRAQELEMEMLSSTSPPERTRYSPIPP

SHHQLTLPDPSHHGLHSNPDSPAKPEKNGHAKIVNPRIAKFFEIQTMPNG

KTRTSLKTMSRRKLSQQKEKKATQMLAIVLGVFIICWLPFFITHILNIHC

DCNIPPVLYSAFTWLGYVNSAVNPIIYTTFNIEFRKAFMKILHC

>cpDRD2 ENSCPOT00000009350 length=439

MDPLNLSWYDDDLERQNWSRPFNGSEGKADRPHYNYYAMLLTLLIFVIVF

GNVLVCMAVSREKALQTTTNYLIVSLAVADLLVAMLVMPWVVYLEVVGEW

KFSRIHCDIFVTLDVMMCTASILNLCAISIDRYTAVAMPMLYNTRYSSKR

RVTVMIAIVWVLSFTISCPMLFGLNNTDQNECIIANPAFVVYSSIVSFYV

PFIVTLLVYIKIYIVLRKRRKRVNTKAFRANLRAPLKANCTHPEDMKLCT

VIMKSNGSFPVNRRRMEAARRAQELEMEIVSSTSPPERTRYSPIPPSHHQ

LTLPAQSHHGFHSTPDSPAKPEKNGHAKDHPKIAKIFEIQSMPNGKTRTS

LKTMSRRKLSQQKEKKATQMLAIVLGVFIICWLPFFITHILNIHCDCNIP

PVLYSAFTWLGYVNSAVNPIIYTTFNIEFRKAFMKILHC

>ocDRD2 XP_002708433.1

MRYLLEEGELALQLVVVKLGILRAWPSSGPAAPMDPLNLSWYDE

DLERQNWSRPLNGSEGRGDRPHYNYYAMLLTLLIFVIVFGNVLVCMAVSREKALQTTT

NYLIVSLAVADLLVATLVMPWVVYLEVVGEWKFSRVHCDIFVTLDVMMCTASILNLCA

ISIDRYTAVAMPMLYNTRYSSKRRVTVMIAIVWVLSLTISCPLLFGLNKTDQNECIIA

NPAFVVYSSIVSFYVPFIVTLLVYIKIYIVLRKRRKRVNTKRSSRAFRANLRAPLKGN

CTHPEDRTLGTVIMKSNGSFPVNRRRVEAARRAQELEMEMLSSTSPPERTRYSPIPPS

HHQLTLPDPSHHGLHSTPDSPAKPEKNGHAKDHPKIAKIFEIQTMPNGKTRTSLKTMS

RRKLSQQKEKKATQMLAIVLGVFIICWLPFFITHILNIHCDCNIPPVLYSAFTWLGYV

NSAVNPIIYTTFNIEFRKAFMKILHC

>moESR1_1 partial

HNDYMCPATNQCTIDKNRRKSCQACRLRKCYEVGMMKGGIRKDR

RGGRMLKHKRQRDDLEGSNGMGSSGDMRAANLWPSPLVIKHTKKNSPALSLTADQMVS

ALLDAEPPLIYSEYDPSRPFSEASMMGLLTNLADRELVHMINWAKRVPGFGDLNLHDQ

VHLLECAWLEILMIGLIWRSMEHPGKLLFAPNLLLDRNQGKCVEGMVEIFDMLLAAST

RFRMMNLQGEEFVCLKSIILLNSGVYTFLSSTLKSLEEKDHIHRVLDKITDTLIHLMA

KAGLTLQQQHRRLAQLLLILSHIRHMSNKGMEHLYNMKCKNVVPLYDLLLEMLDAHRL

HAPASRMGVSPEEPSQSQLATTSSTSAHSLQTYYIPPEAENFPNTI

>moESR1_2 partial

HNDYMCPATNQCTIDKNRRKSCQACRLRKCYEVGMMKGGIRKDR

RGGRMLKHKRQRDDLEGSNGMGSSGDMRAANLWPSPLVIKHTKKNSPALSLTADQMVS

ALLDAEPPLIYSEYDPSRPFSEASMMGLLTNLADRELVHMINWAKRVPGFGDLNLHDQ

VHLLECAWLEILMIGLIWRSMEHPGKLLFAPNLLLD

VYTFLSSTLKSLEEKDHIHRVLDKITDTLIHLMAKAGLTLQQQH

RRLAQLLLILSHIRHMSNKGMEHLYNMKCKNVVPLYDLLLEMLDAHRLHAPASRMGVS

PEEPSQSQLATTSSTSAHSLQTYYIPPEAENFPNTI

>mmuESR1 NP_031982 length=599

MTMTLHTKASGMALLHQIQGNELEPLNRPQLKMPMERALGEVYVDNSKPT

VFNYPEGAAYEFNAAAAAAAAASAPVYGQSGIAYGPGSEAAAFSANSLGA

FPQLNSVSPSPLMLLHPPPQLSPFLHPHGQQVPYYLENEPSAYAVRDTGP

PAFYRSNSDNRRQNGRERLSSSNEKGNMIMESAKETRYCAVCNDYASGYH

YGVWSCEGCKAFFKRSIQGHNDYMCPATNQCTIDKNRRKSCQACRLRKCY

EVGMMKGGIRKDRRGGRMLKHKRQRDDLEGRNEMGASGDMRAANLWPSPL

VIKHTKKNSPALSLTADQMVSALLDAEPPMIYSEYDPSRPFSEASMMGLL

TNLADRELVHMINWAKRVPGFGDLNLHDQVHLLECAWLEILMIGLVWRSM

EHPGKLLFAPNLLLDRNQGKCVEGMVEIFDMLLATSSRFRMMNLQGEEFV

CLKSIILLNSGVYTFLSSTLKSLEEKDHIHRVLDKITDTLIHLMAKAGLT

LQQQHRRLAQLLLILSHIRHMSNKGMEHLYNMKCKNVVPLYDLLLEMLDA

HRLHAPASRMGVPPEEPSQTQLATTSSTSAHSLQTYYIPPEAEGFPNTI

>rnESR1 NP_036821 length=600

MTMTLHTKASGMALLHQIQGNELEPLNRPQLKMPMERALGEVYVDNSKPA

VFNYPEGAAYEFNAAAAAAAAGASAPVYGQSSITYGPGSEAAAFGANSLG

AFPQLNSVSPSPLMLLHPPPHVSPFLHPHGHQVPYYLENEPSAYAVRDTG

PPAFYRSNSDNRRQNGRERLSSSSEKGNMIMESAKETRYCAVCNDYASGY

HYGVWSCEGCKAFFKRSIQGHNDYMCPATNQCTIDKNRRKSCQACRLRKC

YEVGMMKGGIRKDRRGGRMLKHKRQRDDLEGRNEMGTSGDMRAANLWPSP

LVIKHTKKNSPALSLTADQMVSALLDAEPPLIYSEYDPSRPFSEASMMGL

LTNLADRELVHMINWAKRVPGFGDLNLHDQVHLLECAWLEILMIGLVWRS

MEHPGKLLFAPNLLLDRNQGKCVEGMVEIFDMLLATSSRFRMMNLQGEEF

VCLKSIILLNSGVYTFLSSTLKSLEEKDHIHRVLDKINDTLIHLMAKAGL

TLQQQHRRLAQLLLILSHIRHMSNKGMEHLYNMKCKNVVPLYDLLLEMLD

AHRLHAPASRMGVPPEEPSQSQLTTTSSTSAHSLQTYYIPPEAEGFPNTI

>cpESR1 ENSCPOT00000008392 length=596

MTMTLHTKASGMALLHQIQASELEPLSRPQLKMPLEEPQGEVFLDGGKPA

VFNYPEGAAYEFNAAAAASAPVYGQPGLAYGPGSEAAAAFGPSSLGGFPQ

LSSVSPSPLMLLHPPPQLSPFLHPHGQQVPFYLENEPSGYAVREAGAPAF

YRPNSGNRRQSGRERLASTSDKGSITLESAKETRYCAVCNDYASGYHYGV

WSCEGCKAFFKRSIQGHNDYICPATNQCTIDKNRRKSCQACRLRKCYDVG

MIKGGIRKDRRGGRMLKYKRQRDDEERRHEMGPSGDMRGSNLWPSPLVIK

HTKKNSPALSLTADQMVSALMDAEPPLLYSEYDAVKPFSEASMMGLLTNL

ADRELVHMINWAKRVPGFGDLTLHDQVHLLECAWLEILMIGLIWRSMEHP

GKLLFAPNLILDRNQGKCVEGMVEIFDMLLATSTRFRMMNLQGEEFVCLK

SIILLNSGMYTFLSSTLKSLEEKDHIHRVLDKIIDTLIHLMAKAGLTLQQ

QHRRLAQLLLILSHIRHMSNKGVEHLYNMKCKNVVPLYNLLLEMLEAHRL

NTSSNPMGGSPEEPSQSQLATIGSSSAHSLQTYYVSQEAESFPNTI

>ocESR1 gi|291397152|ref|XP_002714993.1| PREDICTED: estrogen receptor alpha-like [Oryctolagus cuniculus]

MTMTLHTKASGMALLHQIQGNELEPLNRPQLKMPLERPLSEVYVDSSKPAVYNYPEGAAYEFNAAAAAAA

AATAPVYSQSGLAYGPGSEAAFGSNGLGGFPQLNSVSPSPLMLLHPPPQLSPFLHPHSQQVPYYLENEPS

GFGVREPGPPAFYRPSSDNRRQGGRERLASTGDKGGMAVESAKETRYCAVCNDYASGYHYGVWSCEGCKA

FFKRSIQGHNDYMCPATNQCTIDKNRRKSCQACRLRKCYEVGMMKGGIRKDRRGGRMLKHKRQRDDGEGR

NETGPPGDMRAANLWPSPLLIKHTKKNSPALSLTADQMVSALLEAEPPIIYSEYDPTRPFSEASMMGLLT

NLADRELVHMINWAKRVPGFVDLTLHDQVHLLECAWLEILMIGLVWRSMEHPGKLLFAPNLLLDRNQGKC

VEGMVEIFDMLLATSSRLRMMNLQGEEFVCLKSIILLNSGVYTFLSSTLKSLEEKDHIHRVLDKITDTLI

HLMAKAGLTLQQQHRRLAQLLLILSHIRHMSNKGMEHLYNMKCKNVVPLYDLLLEMLDAHRLHAPANRGG

VPMEEPAQGQLTTTSAASAHSLQTYYIPGEAEDFPSTI

>moESR2

HNDYICPATNQCTIDKNRRKSCQACRLRKCYEVGMVKCGSRRER

CGYRIVRRQRSSGEQVHCLGKAKRNSGHVPRVKELLLSTLSPEQLVLTLLEAEPPNVL

VSRPSMPFTEASMMMSLTKLADKELVHMIGWAKKIPGFVELSLLDQVRLLESCWMEVL

MVGLMWRSIDHPGKLIFAPDLVLDRSSEDPHWHVAQTKSAAPRDEGKCVEGILEIFDM

LLATTSRFRELKLQHKEYLCVKAMILLNSSMYPLTTASQEAESSRKLTHLLNAVTDAL

VWVIAKSGISSQQQSVRLANLLMLLSHVRHISNKGMEHLLSMKCKNVVPVYDLLLEML

NAHTLGGYKSSLSGSDCSSAEDSKSKEGPQNPQSQ

>mmuESR2 NP_034287 length=549

MSICASSHKDFSQLRPTQDMEIKNSPSSLTSPASYNCSQSILPLEHGPIY

IPSSYVESRHEYSAMTFYSPAVMNYSVPSSTGNLEGGPVRQTASPNVLWP

TSGHLSPLATHCQSSLLYAEPQKSPWCEARSLEHTLPVNRETLKRKLGGS

GCASPVTSPSAKRDAHFCAVCSDYASGYHYGVWSCEGCKAFFKRSIQGHN

DYICPATNQCTIDKNRRKSCQACRLRKCYEVGMVKCGSRRERCGYRIVRR

QRSASEQVHCLNKAKRTSGHTPRVKELLLNSLSPEQLVLTLLEAEPPNVL

VSRPSMPFTEASMMMSLTKLADKELVHMIGWAKKIPGFVELSLLDQVRLL

ESCWMEVLMVGLMWRSIDHPGKLIFAPDLVLDRDEGKCVEGILEIFDMLL

ATTARFRELKLQHKEYLCVKAMILLNSSMYPLATASQEAESSRKLTHLLN

AVTDALVWVISKSGISSQQQSVRLANLLMLLSHVRHISNKGMEHLLSMKC

KNVVPVYDLLLEMLNAHTLRGYKSSISGSECCSTEDSKSKEGSQNLQSQ

>rnESR2 NP_036886 length=485

MTFYSPAVMNYSVPGSTSNLDGGPVRLSTSPNVLWPTSGHLSPLATHCQS

SLLYAEPQKSPWCEARSLEHTLPVNRETLKRKLSGSSCASPVTSPNAKRD

AHFCPVCSDYASGYHYGVWSCEGCKAFFKRSIQGHNDYICPATNQCTIDK

NRRKSCQACRLRKCYEVGMVKCGSRRERCGYRIVRRQRSSSEQVHCLSKA

KRNGGHAPRVKELLLSTLSPEQLVLTLLEAEPPNVLVSRPSMPFTEASMM

MSLTKLADKELVHMIGWAKKIPGFVELSLLDQVRLLESCWMEVLMVGLMW

RSIDHPGKLIFAPDLVLDRDEGKCVEGILEIFDMLLATTSRFRELKLQHK

EYLCVKAMILLNSSMYPLASANQEAESSRKLTHLLNAVTDALVWVIAKSG

ISSQQQSVRLANLLMLLSHVRHISNKGMEHLLSMKCKNVVPVYDLLLEML

NAHTLRGYKSSISGSECSSTEDSKNKESSQNLQSQ

>cpESR2 ENSCPOT00000005764 length=549

MSLCAPSCKEFSQLLPVQDMDIKNSPSSLHSPSSYNCSQSVLPLEHGPLY

IPSSYVDNRHEYSAVTFYSPAVMNYSIPSGVSNTEGVPGRQTTSPSMLWP

APGHLSPLAIHCQSSLLYAEPQKSPWCEARSLEHTLPINRETLKRKVSAG

SCASPAASPASKRDAHFCAVCSDYASGYHYGVWSCEGCKAFFKRSIQGHN

DYICPATNQCTIDKNRRKSCQACRLRKCYEVGMVKCGSRRERCGYRVVRR

QRSPGEQLHCLSKAKKHGGHTPRAKELLLSALSPEQLVLTLLEAEPPNVL

VSRPSAPFTEASMMMSLTKLADKELVHMIGWAKKIPGFVELSLLDQVRLL

ESCWMEVLMVGLMWRSIDHPGKLIFAPDLVLDRDEGKCVEGILEIFDMLL

ATTSRFRELKLQHKEYLCVKAMILLNSNMYPLAAATQEMESSRKLTHLLN

SVTDALVWVIAKSGISSQQQSIRLANLLMLLSHVRHISNKGMEHLLSMKC

KNVVPVYDLLLEMLNAHTLRGYKSPVPGSECSSEEDSKNKEGSQNSQSQ

>ocESR2 ENSOCUT00000005082 length=530

MDIKNSPSSLHSPASYNCSQSVLPLEHGPLYVPSSYVEDHHEYPAMTFYS

PAMMNYSVPSGASEADRGPGRQPTSPDVLWPTPGHLSPLAIRCQSSLLYP

EPQKPSWCEVRPLEHTSPVNREALKRKVSGDSCASPVTSSKKDARFCAVC

SDYASGYHYGVWSCEGCKAFFKRSIQGHNDYICPATNQCTIDKNRRKSCQ

ACRLRKCYEVGMVKCGSRRERCGYRIVRRRRSSGELHCPSRARKNGGPVT

RVKDVLLSALSPEQLVLTLLEAEPPHVLVSRPSSPFTETSMMLSLTKLAD

KELVHMIGWAKKIPGFVELSLLDQVRLLESCWMEVLMVGLIWRSIDHPGK

LIFAPDLVLDRDEGKCVEGILEIFDMLLATTSRFRELKLQHKEYLCVKAM

VLLNSSMCPLAAAAQEAESSRKLTHLLNAVTDALVWVIAKSGISAQQQSV

RLANLLMLLSHVRHASDKGMEHLLSMKCRNVVPVYDLLLEMLNAHTLRGR

RAQRGPRTRSDARPATQAHRGDGSVNPQAV

>moMC4R prt lcl|Sequence 1 ORF:436..1434 Frame +1

MNSTHHHGMYTSLHLWNRSSYGLHGNTSESLGKGYSDGGCYEQLFVSPEVFVTLGVISLLENILVIVAIA

KNKNLHSPMYFFICSLAVADMLVSVSNGSETIVITLLNSTDTDAQSFTVNIDNVIDSVICSSLLASICSL

LSIAVDRYFTIFYALQYHNIMTVKRVAIIISCIWAACTVSGILFIIYSDSSAVIICLITMFFTMLVLMAS

LYVHMFLMARLHIKRIAVLPGTGTIRQGANMKGAITLTILIGVFVVCWAPFFLHLLFYISCPQNPYCVCF

MSHFNLYLILIMCNAVIDPLIYALRSQELRKTFKEIICFYPLGGICDLSGRY

>mmuMC4R gi|85861190|ref|NP_058673.2| melanocortin receptor 4 [Mus musculus]

MNSTHHHGMYTSLHLWNRSSYGLHGNASESLGKGHPDGGCYEQLFVSPEVFVTLGVISLLENILVIVAIA

KNKNLHSPMYFFICSLAVADMLVSVSNGSETIVITLLNSTDTDAQSFTVNIDNVIDSVICSSLLASICSL

LSIAVDRYFTIFYALQYHNIMTVRRVGIIISCIWAACTVSGVLFIIYSDSSAVIICLISMFFTMLVLMAS

LYVHMFLMARLHIKRIAVLPGTGTIRQGTNMKGAITLTILIGVFVVCWAPFFLHLLFYISCPQNPYCVCF

MSHFNLYLILIMCNAVIDPLIYALRSQELRKTFKEIICFYPLGGICELSSRY

>rnMC4R gi|25742776|ref|NP_037231.1| melanocortin receptor 4 [Rattus norvegicus]

MNSTHHHGMYTSLHLWNRSSHGLHGNASESLGKGHSDGGCYEQLFVSPEVFVTLGVISLLENILVIVAIA

KNKNLHSPMYFFICSLAVADMLVSVSNGSETIVITLLNSTDTDAQSFTVNIDNVIDSVICSSLLASICSL

LSIAVDRYFTIFYALQYHNIMTVRRVGIIISCIWAACTVSGVLFIIYSDSSAVIICLITMFFTMLVLMAS

LYVHMFLMARLHIKRIAVLPGTGTIRQGANMKGAITLTILIGVFVVCWAPFFLHLLFYISCPQNPYCVCF

MSHFNLYLILIMCNAVIDPLIYALRSQELRKTFKEIICFYPLGGICELPGRY

>cpMC4R ENSCPOT00000007514 length=333

MMNSTYHHGMHTSLHFWNRSNYGLHSNASEPLGKGYSDGGCYEQLFVSPE

VFVTLGVISLLENILVIVAIAKNKNLHSPMYFFICSLAVADMLVSVSNGS

ETIVITLLNSTDTDAQSFTVNIDNVIDSVICSSLLASICSLLSIAVDRYF

TIFYALQYHNIMTVKRVGIIISCIWAACTVSGILFIIYSDSSAVIICLIT

MFFTMLALMASLYVHMFLMARLHIKRIAVLPGTGTIRQGANMKGAITLTI

LIGVFVVCWAPFFLHLIFYISCPQNPYCVCFMSHFNLYLILIMCNSIIDP

LIYALRSQELRKTFKEIICCYPLGGLCDLSSRY

>ocMC4R ENSOCUT00000022922 length=333

TMNSTHHRGTHTSLHFWNRSSYGLHSNASESLGKDYSDGGCYEQLFVSPE

VFVTLGVISLLENILVIVAIAKNKNLHSPMYFFICSLAVADMLVSISNGS

ETIVITLLNSTDTDAQSFTVNIDNVIDSVICSSLLASICSLLSIAVDRYF

TIFYALQYHNIMTVKRVGIIISCIWAACTVSGILFIIYSDSSAVIICLIT

MFFTMLALMASLYVHMFLMARLHIKRIAVLPGTGAIRQGANMKGAITLTI

LIGVFVVCWAPFFLHLIFYISCPQNPYCVCFMSHFNLYLILIMCNSIIDP

LIYALRSQELRKTFKEIICFYPLGGLCDFSSRY

>moNR3C1_1

MDSKESLAPPAREVPSSLLVRERGNVMDFHKTLRGGATMKVSAS

SSSLAAASQTDSKQQRLLDFPKGSASNAQQPDLSKAVSLSMGLYMGETETKVMGNDLG

YPQQGQISLSSGETDFRLLEESIANLNRSTSVPEKPKSSTSAAGRATPPGKEFPKTHS

DASSEQQNLKSQPGTNGGSVKLYTTDQSTFDILQDLEFSAGSPGKETNQSPWRADLLI

DENLLSPLAGDDDPFLLEGDANEDCKPFILPDTKPKIKDTGDPILPSSSGVTLPQVKT

EKDDFIELCTPGVIKQEKLGPVYCQASFSGTNIIGNKISAISVHGVSTSGGQMYHYDM

NAASLSEQPDQKPVFNVIPPIPVGSENWNRCQGSGEDNLANLGTMNFPARSVFSNGYS

SPGMRPDVSSPPSSSSTAAGPPPKLCLVCSDEASGCHYGVLTCGSCKVFFKRAVEGQH

NYLCAGRNDCIIDKIRRKNCPACRYRKCLQAGMNLEARKTKKKIKGIQQATAGAAQET

SENSNKTVVPATLPQLTPTLVSLLEVIEPEVLYAGYDSSVPDSAWRIMNTLNMLGGRQ

VIAAVKWAKAIPGFRNLHLDDQMTLLQYSWMFLMAFALGWRSYRQASANLLCFAPDLI

INEQRMTLPCMYEQCKQMLYVSSELQRLQVSYEEYLCMKTLLLLSSVPKEGLKSQELF

DEIRMTYIKELGKAIVKREGNSSQNWQRFYQLTKLLDSMHDVVENLLTYCFQTFLDKS

MSIEFPEMLAEIITNQIPKYTNGNIKKLLFHQK

>mmuNR3C1 NP_032199 length=792

MDSKESLAPPGRDEVPSSLLGRGRGSVMDLYKTLRGGATVKVSASSPSVA

AASQADSKQQRILLDFSKGSASNAQQQQQQQQQQQQQQQQQPQPDLSKAV

SLSMGLYMGETETKVMGNDLGYPQQGQLGLSSGETDFRLLEESIANLNRS

TSRPENPKSSTPAAGCATPTEKEFPQTHSDPSSEQQNRKSQPGTNGGSVK

LYTTDQSTFDILQDLEFSAGSPGKETNESPWRSDLLIDENLLSPLAGEDD

PFLLEGDVNEDCKPLILPDTKPKIQDTGDTILSSPSSVALPQVKTEKDDF

IELCTPGVIKQEKLGPVYCQASFSGTNIIGNKMSAISVHGVSTSGGQMYH

YDMNTASLSQQQDQKPVFNVIPPIPVGSENWNRCQGSGEDNLTSLGAMNF

AGRSVFSNGYSSPGMRPDVSSPPSSSSTATGPPPKLCLVCSDEASGCHYG

VLTCGSCKVFFKRAVEGQHNYLCAGRNDCIIDKIRRKNCPACRYRKCLQA

GMNLEARKTKKKIKGIQQATAGVSQDTSENANKTIVPAALPQLTPTLVSL

LEVIEPEVLYAGYDSSVPDSAWRIMTTLNMLGGRQVIAAVKWAKAIPGFR

NLHLDDQMTLLQYSWMFLMAFALGWRSYRQASGNLLCFAPDLIINEQRMT

LPCMYDQCKHMLFISTELQRLQVSYEEYLCMKTLLLLSSVPKEGLKSQEL

FDEIRMTYIKELGKAIVKREGNSSQNWQRFYQLTKLLDSMHDVVENLLSY

CFQTFLDKSMSIEFPEMLAEIITNQIPKYSNGNIKKLLFHQK

>rnNR3C1 NP_036708 length=794

MDSKESLAPPGRDEVPGSLLGQGRGSVMDFYKSLRGGATVKVSASSPSVA

AASQADSKQQRILLDFSKGSTSNVQQRQQQQQQQQQQQQQQQQQQPDLSK

AVSLSMGLYMGETETKVMGNDLGYPQQGQLGLSSGETDFRLLEESIANLN

RSTSVPENPKSSTSATGCATPTEKEFPKTHSDASSEQQNRKSQTGTNGGS

VKLYPTDQSTFDLLKDLEFSAGSPGKDTNESPWRSDLLIDENLLSPLAGE

DDPFLLEGDTNEDCKPLILPDTKPKIKDTGDTILSSPSSVALPQVKTEKD

DFIELCTPGVIKQEKLGPVYCQASFSGTNIIGNKMSAISVHGVSTSGGQM

YHYDMNTASLSQQQDQKPVFNVIPPIPVGSENWNRCQGSGEDSLTSLGAL

NFPGRSVFSNGYSSPGMRPDVSSPPSSSSAATGPPPKLCLVCSDEASGCH

YGVLTCGSCKVFFKRAVEGQHNYLCAGRNDCIIDKIRRKNCPACRYRKCL

QAGMNLEARKTKKKIKGIQQATAGVSQDTSENPNKTIVPAALPQLTPTLV

SLLEVIEPEVLYAGYDSSVPDSAWRIMTTLNMLGGRQVIAAVKWAKAIPG

FRNLHLDDQMTLLQYSWMFLMAFALGWRSYRQSSGNLLCFAPDLIINEQR

MSLPCMYDQCKHMLFVSSELQRLQVSYEEYLCMKTLLLLSSVPKEGLKSQ

ELFDEIRMTYIKELGKAIVKREGNSSQNWQRFYQLTKLLDSMHEVVENLL

TYCFQTFLDKTMSIEFPEMLAEIITNQIPKYSNGNIKKLLFHQK

>cpNR3C1 ENSCPOT00000024687 length=771

MDLKESVTSSKEVPSSVLGSERRNVIDFYKTVRGGATVKVSASSPSLAAA

AQSDSKQRRLLVDFPKGSGSNAQQPDLSKAVSLSMGLYMGETETKVMGND

LGFPQQGQISLPSGETDFRLLEESIANLSRSTSVPENPKNSASAVSGTPT

EEFPKTQSDLSSEQQNLKSQAGTNGGNVKFPPDQSTFDILKDLEFSSGSP

GKERSESPWRPDLLMDESCLLSPLAGEDDPFLLEGNSNEDCKPLILPDTK

PKIKDNGDGILSSSNSVPQPQVKMEKEDFIELCTPGVIKQEKLGPVYCQA

SFSGANIIGNKMSAISVHGVSTSGGQMYHYDMNTASLSQQQDQKPIFNVI

PPIPVGSENWNRCQGSGEDNLTSLGTVNFPGRSVFSNGYSSPGLRPDVSS

PPSSSSTTTGPPPKLCLVCSDEASGCHYGVLTCGSCKVFFKRAVEGQHNY

LCAGRNDCIIDKIRRKNCPACRYRKCLQAGMNLEGNKNSVRIRGIQQATT

GVSQNTSENPNKTIVPATLPQLTPTLVSLLEVIEPEVIHSGYDSTSPDST

WRIMTTLNMLGGRQVIAAVKWAKAIPGFKNLHLDDQMTLLQYSWMFLMAF

ALGWRSYKQSNGSLLCFAPDLIINEQRMSLPWMYDQCRYMLYVSSELKRL

QVSYEEYLCMKTLLLLSSVPKEGLKSQELFDEIRMTYIKELGKAIVKREG

NSSQNWQRFYQLTKLLDSLHEIVGNLLNICFKTFLDKTMNIEFPEMLAEI

ITNQLPKYSNGDIKKLLFHQK

>ocNR3C1 NP_001075616 length=772

MDSKESLSPPGREEVPSSVLRPAERGNVMDLYKTLRGGAPVRVPASSPSL

APAAQPDSKQQRLAVDFPKGSASNAQQPDLSRAVSLSMGLYMGETETKVM

GSDLAFPQQGQTSLSSGETDFRLLEESIASLNRSASGADNPRSTAPAAGS

AAPTEGFPKTHSDLASERQNPKGQTGGSAGSAKLHPTDQSTFDILQDLEF

SGSPSKDRSESPWRSDLLMDENCLLSPLAGEDDPFLLEGNSSEDCKPLIL

PDTKPKIKDNGDLILSNSNNVPLPQVKTEKEDFIELCTPGVIKQEKLGPV

YCQASFSGANIIGNKISAISVHGVSTSGGQMYHYDMNAQQQEQKPLFNVI

PPIPVGSENWNRCQGSGDDNLTSLGTMNFPGRSVFSNGYSSPGMRPDVSS

PPSNSTTAAGPPPKLCLVCSDEASGCHYGVLTCGSCKVFFKRAVKGQHNY

LCAGRNDCIIDKIRRKNCPACRYRKCLQAGMNLEARKTKKKIKGIQQTST

GVSQETSENPSNRTVVPAALPQLTPTLVSLLEVIEPEVLYAGYDSSVPDS

TWRIMTTLNMLGGRQVIAAVKWAKAIPGFRNLHLDDQMTLLQYSWMFLMA

FALGWRSYKQSSGNMLCFAPDLVINEQRMTLPYMYDQCKHMLFVSSELKR

LQVSYEEYLCMKTLLLLSTVPKEGLKSQELFDEIRMTYIKELGKAIVKRE

GNSSQNWQRFYQLTKLLDSMHEVVENLLHYCFQTFLDKTMSIEFPEMLAE

IITNQIPKYSNGNIKKLLFHQK

>moNTRK2

MSPWPRWHGPAMARLWGLCWLAVGFWRASLACPMSCKCSASRIW

CTEPSPGIVAFPRLEPNSVDPENITEIFIANQKRLEIINEDDVEAYVGLKNLTIVDSG

LKFVAYKAFLKNSNLQHVNLTRNKLTSLSRKHFRHLDLSELVLVGNPFTCSCDIMWIK

TLQETKGSPDTQDLYCLNESSKNTPLANLQIPNCGLPSARLAAPNLTVEEGNSVTLSC

SVAGDPLPTLYWDVGNLVSKHMNETSHTQGSLRITNISSDDSGKQISCVAENLVGEDQ

DSVNLTVHFAPSITFLESPTSDHHWCIPFTVRGNPKPTLQWFYNGAILNESKYICTKI

HVTNHTEYHGCLQLDNPTHMNNGDYTLMAKNEYGKDERQISAHFMGRPGIDYETNPNY

PEDLYEDWTVATDIGDTTNKSNEIPSTDVADQNNRERLSVYAVVVIASVVGFCLLVML

LLLKLARHSKFGMKGFVLFHKIPLDG

>mmuNTRK2 ENSMUST00000079828 length=821

MSPWLKWHGPAMARLWGLCLLVLGFWRASLACPTSCKCSSARIWCTEPSP

GIVAFPRLEPNSVDPENITEILIANQKRLEIINEDDVEAYVGLRNLTIVD

SGLKFVAYKAFLKNSNLRHINFTRNKLTSLSRRHFRHLDLSDLILTGNPF

TCSCDIMWLKTLQETKSSPDTQDLYCLNESSKNMPLANLQIPNCGLPSAR

LAAPNLTVEEGKSVTLSCSVGGDPLPTLYWDVGNLVSKHMNETSHTQGSL

RITNISSDDSGKQISCVAENLVGEDQDSVNLTVHFAPTITFLESPTSDHH

WCIPFTVRGNPKPALQWFYNGAILNESKYICTKIHVTNHTEYHGCLQLDN

PTHMNNGDYTLMAKNEYGKDERQISAHFMGRPGVDYETNPNYPEVLYEDW

TTPTDIGDTTNKSNEIPSTDVADQSNREHLSVYAVVVIASVVGFCLLVML

LLLKLARHSKFGMKGPASVISNDDDSASPLHHISNGSNTPSSSEGGPDAV

IIGMTKIPVIENPQYFGITNSQLKPDTFVQHIKRHNIVLKRELGEGAFGK

VFLAECYNLCPEQDKILVAVKTLKDASDNARKDFHREAELLTNLQHEHIV

KFYGVCVEGDPLIMVFEYMKHGDLNKFLRAHGPDAVLMAEGNPPTELTQS

QMLHIAQQIAAGMVYLASQHFVHRDLATRNCLVGENLLVKIGDFGMSRDV

YSTDYYRVGGHTMLPIRWMPPESIMYRKFTTESDVWSLGVVLWEIFTYGK

QPWYQLSNNEVIECITQGRVLQRPRTCPQEVYELMLGCWQREPHTRKNIK

SIHTLLQNLAKASPVYLDILG

>rnNTRK2 ENSRNOT00000025461 length=772

MSPWPRWHGPAMARLWGLCLLVLGFWRASLACPMSCKCSTTRIWCTEPSP

GIVAFPRLEPNSIDPENITEINFTRNKLTSLSRRHFRHLDLSDLILTGNP

FTCSCDIMWLKTLQETKSSPDTQDLYCLNESSKNTPLANLQIPNCGLPSA

RLAAPNLTVEEGKSVTISCSVGGDPLPTLYWDVGNLVSKHMNETSHTQGS

LRITNISSDDSGKQISCVAENLVGEDQDSVNLTVHFAPTITFLESPTSDH

HWCIPFTVRGNPKPALQWFYNGAILNESKYICTKIHVTNHTEYHGCLQLD

NPTHMNNGDYTLMAKNEYGKDERQISAHFMGRPGVDYETNPNYPEVLYED

WTTPTDIGDTTNKSNEIPSTDVADQTNREHLSVYAVVVIASVVGFCLLVM

LLLLKLARHSKFGMKGPASVISNDDDSASPLHHISNGSNTPSSSEGGPDA

VIIGMTKIPVIENPQYFGITNSQLKPDTFVQHIKRHNIVLKRELGEGAFG

KVFLAECYNLCPEQDKILVAVKTLKDASDNARKDFHREAELLTNLQHEHI

VKFYGVCVEGDPLIMVFEYMKHGDLNKFLRAHGPDAVLMAEGNPPTELTQ

SQMLHIAQQIAAGMVYLASQHFVHRDLATRNCLVGENLLVKIGDFGMSRD

VYSTDYYRVGGHTMLPIRWMPPESIMYRKFTTESDVWSLGVVLWEIFTYG

KQPWYQLSNNEVIECITQGRVLQRPRTCPQEVYELMLGCWQREPHTRKNI

KNIHTLLQNLAKASPVYLDILG

>cpNTRK2 scaffold_11.147.1 length=805

MARLWGICWLILGFWRSALACPQPCKCSASRIWCSNPSPGIVAFPRLEPN

SADPENITEIFIANQKRLEIINEDDVEAYVGLRNLTIVDSGLKFVAHKAF

LKNSNLQHINLTRNKLTSLSRKHFQHLDLSELILVGNPFVCSCDIMWIKT

LQEAKSSPETQDLYCLNESSKNIPLANLQIPNCGLPSVNLIAPNLTVEEG

KSITLSCRVAGDPLPNMYWDVGNLVSKHMNETNQTQGSLKITNISADDSG

KQISCVAENLVGEDQDSVNLTVHFAPTITFLESPTSDHHWCIPFTVKGNP

KPALQWFYNGAILNESKYICTKIHVTNHTEYHGCLQLDNPTHMNNGDYTL

IAQNEYGKDERQVSAHFMGWPGDIDGGNPDYLGELYEDWTATSDTTNRSS

EMPSTVDEPGREHLSVYAVVVIASVVGFCLLVMLFLLKLARHSKFGMKGP

ASVISNDDDSASPLHHISNGSNTPSSSEGGPDAVIIGMTKIPVIENPQYF

GITNSQLKPDTFVQHIKRHNIVLKRELGEGAFGKVFLAECYNLCPEQDKI

LVAVKTLKDASDNARKDFHREAELLTNLQHEHIVKFYGVCVEGDPLIMVF

EYMKHGDLNKFLRAHGPDAVLMAEGNPPTELTQSQMLHIAQQIAAGMVYL

ASQHFVHRDLATRNCLVGENLLVKIGDFGMSRDVYSTDYYRVGGHTMLPI

RWMPPESIMYRKFTTESDVWSLGVVLWEIFTYGKQPWYQLSNNEVIECIT

QGRVLQRPRTCPQEVYELMLGCWQREPHMRKSIKGIHTLLQNLAKASPVY

LDILG

>ocNTRK2 ENSOCUT00000009741 length=838

MSLWTRWHGPTMARLWSFCWLALGLWRPAVACPASCTCSASRIWCSDPVP

GLMAFPRLEPNSADPENITEIFIANQKKLEIINEDDIEAYVGLRNLTIVD

SGLKFVAYKAFLKNSNLQHINFTRNKLTSLSRKHFRHLDLSELILVGNPF

TCSCDIMWIKTLQETKSSPETQDLYCLNESSKNIPLANLQIPNCGLPSAS

LAAPNLTVEEGKSITLSCNVAGDPVPNLYWDVGNLVSKHMNETSHTQGFL

RITNISSDDSGKQISCVAENLVGEDQDSVNLTVHFAPTITFLESPTSDHH

WCIPFTVKGNPKPTLQWFYNGAILNESKYICTKIHVTNHTEYHGCLQLDN

PTHMNNGDYTLIAKNEYGKDERQISAHFMGWPGIDDDPNLNYPDVIYADY

GTAANDIGDTTNRSNEIPPTGAADNAGREHLSVYAVVVIASVVGFCLLVM

LFLLKLARHSKFGMKDFSWFGFGKVKSRQGVGPASVISNDDDSASPLHHI

SNGSNTPSSSEGGPDAVIIGMTKIPVIENPQYFGITNSQLKPDTFVQHIK

RHNIVLKRELGEGAFGKVFLAECYNLCPEQDKILVAVKTLKDASDNARKD

FHREAELLTNLQHEHIVKFYGVCVEGDPLIMVFEYMKHGDLNKFLRAHGP

DAVLMAEGTPPTELTQSQMLHIAQQIAAGMVYLASQHFVHRDLATRNCLV

GENLLVKIGDFGMSRDVYSTDYYRVGGHTMLPIRWMPPESIMYRKFTTES

DVWSLGVVLWEIFTYGKQPWYQLSNNEVIECITQGRVLQRPRTCPQEVYE

LMLGCWQREPHMRKSIKDIHTLLQNLAKASPVYLDILG

>moOXT

MACPSLACCLLGLLALTSACYIQNCPLGGKRAALDLDTRKCLPC

GPGGKGRCFGPNICCADELGCFVGTAEALRCQEENYLPSPCQSGQKPCGSGGRCAAAG

VCCSPDGCRMDPACDPESAFSER

>mmuOXT ENSMUST00000028764 length=125

MACPSLACCLLGLLALTSACYIQNCPLGGKRAVLDLDMRKCLPCGPGGKG

RCFGPSICCADELGCFVGTAEALRCQEENYLPSPCQSGQKPCGSGGRCAA

TGICCSPDGCRTDPACDPESAFSER

>rnOXT ENSRNOT00000028829 length=125

MACPSLACCLLGLLALTSACYIQNCPLGGKRAALDLDMRKCLPCGPGGKG

RCFGPSICCADELGCFVGTAEALRCQEENYLPSPCQSGQKPCGSGGRCAT

AGICCSPDGCRTDPACDPESAFSER

>cpOTX ENSCPOT00000001738 length=125

MAGPSLACCLLGLLALTSACYIQNCPLGGKRAALDLDVRKCLPCGPGGQG

RCFGPSICCADALGCFVGTAEALRCQEENYLPSPCQSGQKPCGSGGRCAA

NGVCCNDDGCRIDPACDSEAAFAER

>ocOXT ENSOCUT00000008351 length=128

SRTMAGPSLACCLLGLLALTSACYIQNCPLGGKRAALDRDVRKCLPCGPA

GKGRCFGPSICCADELGCFVGTAEALRCQEENFLPSPCQSGQKPCGSGGR

CAAAGVCCSADGCRTDPTCDPEAAFSER

>moOXTR

MEGTPAANWSFELDLGSGVSPGVEGNLTAGPPQRNEALARVEVA

VLCLILFLALSGNACVLLALRITRHKHSRLFFFMKHLSIADLVVAVFQVLPQLLWDIT

FRFYGPDLLCRLVKYLQVVGMFASTYLLLLMSLDRCLAICQPLRSLRRRTDRLAVLAT

WLGCLVASAPQVHIFSLREVADGVFDCWAVFIQPWGPKAYVTWITLAVYIVPVIVLAA

CYGLISFKIWQNLRLKTAAAAAEGTEGSAAGGAGRAALARVSSVKLISKAKIRTVKMT

FIIVLAFIVCWTPFFFVQMWSVWDVNAPKEASAFIIAMLLASLNSCCNPWIYMLFTGH

LFHELVQRFLCCSARYLKGSRPGETSVSKKSNSSTFVLSRRSSSQRSCSQPSSA

>mmuOXTR NP_001074616 length=388

MEGTPAANWSIELDLGSGVPPGAEGNLTAGPPRRNEALARVEVAVLCLIL

FLALSGNACVLLALRTTRHKHSRLFFFMKHLSIADLVVAVFQVLPQLLWD

ITFRFYGPDLLCRLVKYLQVVGMFASTYLLLLMSLDRCLAICQPLRSLRR

RTDRLAVLATWLGCLVASVPQVHIFSLREVADGVFDCWAVFIQPWGPKAY

VTWITLAVYIVPVIVLAACYGLISFKIWQNLRLKTAAAAAAAEGSDAAGG

AGRAALARVSSVKLISKAKIRTVKMTFIIVLAFIVCWTPFFFVQMWSVWD

VNAPKEASAFIIAMLLASLNSCCNPWIYMLFTGHLFHELVQRFLCCSARY

LKGSRPGETSISKKSNSSTFVLSRRSSSQRSCSQPSSA

>rnOXTR NP_037003 length=388

MEGTPAANWSVELDLGSGVPPGEEGNRTAGPPQRNEALARVEVAVLCLIL

FLALSGNACVLLALRTTRHKHSRLFFFMKHLSIADLVVAVFQVLPQLLWD

ITFRFYGPDLLCRLVKYLQVVGMFASTYLLLLMSLDRCLAICQPLRSLRR

RTDRLAVLGTWLGCLVASAPQVHIFSLREVADGVFDCWAVFIQPWGPKAY

VTWITLAVYIVPVIVLAACYGLISFKIWQNLRLKTAAAAAAAEGNDAAGG

AGRAALARVSSVKLISKAKIRTVKMTFIIVLAFIVCWTPFFFVQMWSVWD

VNAPKEASAFIIAMLLASLNSCCNPWIYMLFTGHLFHELVQRFFCCSARY

LKGSRPGETSVSKKSNSSTFVLSRRSSSQRSCSQPSSA

>ocOXTR ENSOCUT00000005950 length=393

MEGALVANWSTEAVGASAVSPGTTENGTVGPPQRNEALARVEVAVLCLIL

FLALSGNVCVLLALRTTRHKHSRLFFFMKHLSIADLVVAVFQVLPQLLWD

ITFRFYGPDLLCRLVKYLQVVGMFASTYMLLLMSLDRCLAICRPLRTLRR

RTDRLAVLATWLGCLVASAPQVHIFSLREVAEGVFDCWAVFIQPWGPKAY

VTWITLAVYIVPVIVLAACYGLISFKIWQNLRLKTLAAAAAAAGPEGAAA

AAAAGGAERAALARVSSVKLISKAKIRTVKMTFIIVLAFIVCWTPFFFVQ

MWSVWDADAPKEASAFIIAMLLASLNSCCNPWIYLLFTGHLFQELVQRFL

CCSPGYRRGGRQGETSVSKKSNSSTFVLSRRSSSQRSCSQPTT

>cpOXTR ENSCPOT00000019629 length=390

MAGALAANWSAEDAANASAVLPGSERNSTAGPPQRNEALARVEVAVLCVI

LFLALSGNACVLLALRTTRHKHSRLFFFMKHLSIADLVVAVFQVLPQLLW

DITFRFYGPDLLCRLVKYLQVVGMFASTYLLLLMSLDRCLAICQPLRSLR

RRTDRLAVLATWLGCLVASVPQVHIFSLREVAEGVFDCWAVFIQPWGPKA

YVTWITLAVYIVPVIVLAACYGLISFKIWQNLKLKTAAEAAAQGPEDSAT

AGTERAALARVSNVKLISKAKIRTVKMTFIVVLAFIVCWTPFFFVQMWSV

WDADAPKEASAFIIAMLLASLNSCCNPWIYMLFTGHLFHELVQRFLCCYP

RHLRGSWPGETSISRKSNSSTFVLSQRSSSQRSCSQPSTA

>moSLC6A2

MLLARMNPQVQPELGGADPLPEQPLRPCKTADLLVVKERNGVQC

LLASQDGDAQPRETWGKKIDFLLSVVGFAVDLANVWRFPYLCYKNGGGAFLIPYTLFL

IIAGMPLFYMELALGQYNREGAATVWKICPFFKGVGYAVILIALYVGFYYNVIIAWSL

YYLFASFTLNLPWTNCGHAWNSPNCTDPKLLNASVLGDHTKYSKYKFTPAAEFYERGV

LHLHESSGIHDIGLPQWQLLLCLMVVIIVLYFSLWKGVKTSGKVVWITATLPYFVLFV

LLVHGVTLPGASNGINAYLHIDFYRLKEATVWIDAATQIFFSLGAGFGVLIAFASYNK

FDNNCYRDALLTSTINCVTSFISGFAIFSILGYMAHEHKVKIEDVATEGAGLVFVLYP

EAISTLSGSTFWAVLFFLMLLALGLDSSMGGMEAVITGLADDFQVLKRHRKLFTFGVT

IGTFLLAMFCITKGGIYVLTLLDTFAAGTSILFAVLMEAIGVSWFYGVDRFSNDIQQM

MGFKPGLYWRLCWKFVSPAFLLFVVVVSIINFKPLTYDDYVYPPWANWVGWGIALSSM

ILVPAYVIYKFFSIRGSLWERVAYGITPENEHHLVAQRDVRQFQLRHWLAI

>mmuSLC6A2 ENSMUST00000072939 length=617

MLLARMNPQVQPELGGADPLPEQPLRPCKTADLLVVKERNGVQCLLASQD

SDAQPRETWGKKIDFLLSVVGFAVDLANVWRFPYLCYKNGGGAFLIPYTL

FLIIAGMPLFYMELALGQYNREGAATVWKICPFFKGVGYAVILIALYVGF

YYNVIIAWSLYYLFASFTLNLPWTNCGHSWNSPNCTDPKLLNASVLGDHT

KYSKYKFTPAAEFYERGVLHLHESSGIHDIGLPQWQLLLCLMVVIVVLYF

SLWKGVKTSGKVVWITATLPYFVLFVLLVHGVTLPGASNGINAYLHIDFY

RLKEATVWIDAATQIFFSLGAGFGVLIAFASYNKFDNNCYRDALLTSTIN

CVTSFISGFAIFSILGYMAHEHKVNIEDVATEGAGLVFILYPEAISTLSG

STFWAVLFFLMLLALGLDSSMGGMEAVITGLADDFQVLKRHRKLFTCVVT

ISTFLLALFCITKGGIYVLTLLDTFAAGTSILFAVLMEAIGVSWFYGVDR

FSNDIQQMMGFKPGLYWRLCWKFVSPAFLLFVVVVSIINFKPLTYDDYTY

PPWANWVGWGIALSSMILVPAYVIYKFLSIRGSLWERVAYGITPENEHHL

VAQRDVRQFQLRHWLAI

>rnSLC6A2 ENSRNOT00000022285 length=597

MVTRSRETWGKKIDFLLSVVGFAVDLANVWRFPYLCYKNGGGAFLIPYTL

FLIIAGMPLFYMELALGQFNREGAATVWKICPFFKGVGYAVILIALYVGF

YYNVIIAWSLYYLFASFTLNLPWTNCGHAWNSPNCTDPKLLNASVLGDHT

KYSKYKFTPAAEFYERGVLHLHESSGIHDIGLPQWQLLLCLMVVIVVLYF

SLWKGVKTSGKVVWITATLPYFVLFVLLVHGVTLPGASNGINAYLHIDFY

RLKEATVWIDAATQIFFSLGAGFGVLIAFASYNKFDNNCYRDALLTSTIN

CVTSFISGFAIFSILGYMAHEHKVKIEDVATEGAGLVFVLYPEAISTLSG

STFWAVLFFLMLLALGLDSSMGGMEAVITGLADDFQVLKRHRKLFTCAVT

LGTFLLAMFCITKGGIYVLTLLDTFAAGTSILFAVLMEAIGVSWFYGVDR

FSNDIQQMMGFKPGLYWRLCWKFVSPAFLLFVVVVSIINFKPLTYDDYVY

PPWANWVGWGIALSSMILVPAYVIYKFFSIRGSLWERVAYGITPENEHHL

LALEIELSSLQSFVITSCPIDPLLSSFLFISCQKTLVFKKSGPAPLI

>cpSLC6A2 ENSCPOT00000002303 length=617

ILLARMNPQVQPENSGADPESLQPSRGCKTADLLVVKEHNGVQCLLAPRD

RDAQPRETWGKKIDFLLSVVGFAVDLANVWRFPFLCYKNGGGAFLIPYTL

FLIIAGMPLFYMELALGQYNREGAATVWKICPFFKGVGYAVILMALYVGF

YYNAIIAWSFYYLFASFTLSLPWTDCGHAWNSPNCTDPKLFNSSVLGNHT

KYSKYKFTPAAEFYERGVLHLHESDGIHDIGLPQWQLLLCLMLVVIILYF

SLWKGVKTSGKVVWITATLPYFVLFVLLVHGITLPGASNGISAYLHIDFY

RLKDATVWIDAATQIFFSLGAGFGVLVAFASYNKFDNNCYRDAMLTSTIN

CVTSFISGFAIFSILGYMAHEHKVNIEDVATEGAGLVFILYPEAISTLSG

STFWAILFFIMLLTLGLDSSMGGMEAVITGLADDFQVLKRHRKLFTLGVT

LGTFLLALFCITKGGIYVLTLLDTFAAGTSILFAVLIEAIGVSWFYGVDR

FSNDIQQMMGFKPGLYWRLCWKFFSPAFLLFVVVVSIVNFKPLTYDDYVF

PTWANWLGWAISLSSMALVPAYIIYKCLSTRGSLRERLAYGITPENEHHL

VAQREIRQFQVQHWLAI

>ocSLC6A2 ENSOCUT00000017313 length=617

MLLARMNPQVQPENSGADPGPQPPLKACKAADLVVVKDSNGVQCLLAPRG

DDAQPRETWGKKMDFLLSVVGFAVDLANVWRFPYLCYKNGGGAFLIPYTL

FLIIAGMPLFYMELALGQYNREGAATVWKICPFFKGVGYAVILIALYVGF

YYNVIIAWSLYYLFSSFTFNLPWTDCGHAWNSPNCTDPKLLNGSVLGNHT

KYSKYRFTPAAEFYERGVLHLHESSGIHDIGLPQWQLLLCLMVVVIVLYF

SLWKGVKTSGKVVWITATLPYLVLFVLLVHGVTLPGASNGINAYLHIDFY

RLKEATVWIDAATQIFFSLGAGFGVLIAFASYNKFDNNCYRDALLTSTIN

CVTSFFSGFAIFSILGYMAHEHKVNIEDVATEGAGLVFILYPEAISTLSG

STFWAIVFFIMLLALGFDSSMGGMEAVITGLADDFQVLKRHRKLFTLAVT

IGTFLLALFCITKGGIYVVTLLDTFAAGTSILFAVLMEAIGVSWFYGVDR

FSNDIQQMMGFKPGLYWRLCWKFVSPAFLLFVVVVSIVNFKPLTYDDYVF

PPWANWMGWGIALSSMVLVPSYVIYKFLSIPGSLWERLAYGITPENEHHL

VAQRDVKQFQLQHWLAI

>moSLC6A3_1

AFLVPYLLFMVIAGMPLFYMELALGQFNREGAAGVWKICPILKG

VGFTVILISFYVGFFYNVIIAWALHYFFSSFTMDLPWIHCNNTWNSPNCSDAHASNSS

DGLGLNDTFGTTPAAEYFERGVLHLHQSSGIDDLGPPRWQLTACLVLVIVLLYFSLWK

GVKTSGKVVWITATMPYVVLTALLLRGVTLPGALDGIKAYLSVDFYRLCEASVWIDAA

TQVCFSLGIGFGVLIAFSSYNKFTNNCYRDAIITTSINSLTSFSSGFVVFSFLGYMAQ

KHNVPIGDVATDGPGLIFIIYPEAIATLPLSSAWAAVFFLMLLTLGIDSAMGGMESVI

TGLVDEFQLLHRHRELFTLGIVLATFLLSLFCVTNGGIYVFTLLDHFAAGTSILFGVL

IEAIGVAWFYGVKQFSDDIKQMTGQRPNLYWRLCWKLVSPCFLLYVVVVSIVTFRPPH

YGDYIFPDWANALGWIIATSSMAMVPIYATYKFCSLPGSFREKLAYAITPEKDRQLVD

RGEVRQFTLRHWLLV

>moSLC6A3_2

VGFTVILISFYVGFFYNVIIAWALHYFFSSFTMDLPWIHCNNTW

NSPNCSDAHASNSSDGLGLNDTFGTTPAAEYFERGVLHLHQSSGIDDLGPPRWQLTAC

LVLVIVLLYFSLWKGVKTSGKVVWITATMPYVVLTALLLRGVTLPGALDGIKAYLSVD

FYRLCEASVWIDAATQVCFSLGIGFGVLIAFSSYNKFTNNCYRDAIITTSINSLTSFS

SGFVVFSFLGYMAQKHNVPIGDVATDGPGLIFIIYPEAIATLPLSSAWAAVFFLMLLT

LGIDSAMGGMESVITGLVDEFQLLHRHRELFTLGIVLATFLLSLFCVTNGGIYVFTLL

DHFAAGTSILFGVLIEAIGVAWFYGVKQFSDDIKQMTGQRPNLYWRLCWKLVSPCFLL

YVVVVSIVTFRPPHYGDYIFPDWANALGWIIATSSMAMVPIYATYKFCSLPGSFREKL

AYAITPEKDRQLVDRGEVRQFTLRHWLLV

>mmuSLC6A3 ENSMUST00000022100 length=619

MSKSKCSVGPMSSVVAPAKEPNAVGPREVELILVKEQNGVQLTNSTLINP

PQTPVEVQERETWSKKIDFLLSVIGFAVDLANVWRFPYLCYKNGGGAFLV

PYLLFMVIAGMPLFYMELALGQFNREGAAGVWKICPVLKGVGFTVILISF

YVGFFYNVIIAWALHYFFSSFTMDLPWIHCNNTWNSPNCSDAHSSNSSDG

LGLNDTFGTTPAAEYFERGVLHLHQSRGIDDLGPPRWQLTACLVLVIVLL

YFSLWKGVKTSGKVVWITATMPYVVLTALLLRGVTLPGAMDGIRAYLSVD

FYRLCEASVWIDAATQVCFSLGVGFGVLIAFSSYNKFTNNCYRDAIITTS

INSLTSFSSGFVVFSFLGYMAQKHNVPIRDVATDGPGLIFIIYPEAIATL

PLSSAWAAVFFLMLLTLGIDSAMGGMESVITGLVDEFQLLHRHRELFTLG

IVLATFLLSLFCVTNGGIYVFTLLDHFAAGTSILFGVLIEAIGVAWFYGV

QQFSDDIKQMTGQRPNLYWRLCWKLVSPCFLLYVVVVSIVTFRPPHYGAY

IFPDWANALGWIIATSSMAMVPIYATYKFCSLPGSFREKLAYAITPEKDR

QLVDRGEVRQFTLRHWLLV

>rnSLC6A3 ENSRNOT00000040291 length=619

MSKSKCSVGPMSSVVAPAKESNAVGPREVELILVKEQNGVQLTNSTLINP

PQTPVEAQERETWSKKIDFLLSVIGFAVDLANVWRFPYLCYKNGGGAFLV

PYLLFMVIAGMPLFYMELALGQFNREGAAGVWKICPVLKGVGFTVILISF

YVGFFYNVIIAWALHYFFSSFTMDLPWIHCNNTWNSPNCSDAHASNSSDG

LGLNDTFGTTPAAEYFERGVLHLHQSRGIDDLGPPRWQLTACLVLVIVLL

YFSLWKGVKTSGKVVWITATMPYVVLTALLLRGVTLPGAMDGIRAYLSVD

FYRLCEASVWIDAATQVCFSLGVGFGVLIAFSSYNKFTNNCYRDAIITTS

INSLTSFSSGFVVFSFLGYMAQKHNVPIRDVATDGPGLIFIIYPEAIATL

PLSSAWAAVFFLMLLTLGIDSAMGGMESVITGLVDEFQLLHRHRELFTLG

IVLATFLLSLFCVTNGGIYVFTLLDHFAAGTSILFGVLIEAIGVAWFYGV

QQFSDDIKQMTGQRPNLYWRLCWKLVSPCFLLYVVVVSIVTFRPPHYGAY

IFPDWANALGWIIATSSMAMVPIYATYKFCSLPGSFREKLAYAITPEKDH

QLVDRGEVRQFTLRHWLLL

>cpSLC6A3 ENSCPOT00000026455 length=619

MSGRRCPVGLTSSVVAPAKEPNAMGPREVELVLVKEQNGVQLTNSTLISA

PHSPTGAQERETWGKKIDFLLSVIGFAVDLANVWRFPYLCYKNGGGAFLV

PYLLFMVIAGMPLFYMELALGQFNREGAAGVWKICPILKGVGFTVILISL

YVGFFYNVIIAWALHYFFSSFSVELPWTHCNNTWNSPNCSDAPSGNASTG

PGLNSTFGTTPAAEYFERGVLHLHLSHGIDDLGPPRWQLTACLVLVIVLL

YFSLWKGVKTSGKVVWITATMPYVVLTALLLRGLTLPGAIDGIRAYLSVD

FYRLCEASVWIDAATQVCFSLGVGFGVLIAFSSYNQFTNNCYRDAILTTS

INSLTSFSSGFVVFSFLGYMAQKHSVPIGDVAKDGPGLIFIIYPEAIATL

PLSSAWAVVFFIMLLTLGLDSAMGGMESVITGLIDEFQLLHRHRELFTLC

IVLGTFLLSLFCVTNGGIYVFTLLDHFAAGTSILFGVLIEAIGVAWFYGV

RQFSEDIKQMTGRRPSLYWRLCWKLVSPCFLLFVVVVSVVTFRPPHYGSY

VFPAWANALGWAVAASSMAMVPIYAAYKFCSLPGSFREKLAQAITPEKDR

ELVNRGEVRQFTLRHWLLV

>ocSLC6A3 ENSOCUT00000026679 length=313

APLAPGVGFTVILISLYVGFFYNVIIAWALHYLFSSFAAELPWTHCNNTW

NSPSCSDARHGRASHGNASHGRAGHDASLNHTFGTTPAAEYFERGVLHLH

QSHGIDDLGPPRWELTACLVLVITLLYFSLWKGVKTSGKVVWITATMPYV

VLTALLLRGLTLPGALDGIRAYLSVDFRRLCEASVWIDAATQVCFSLGVG

FGVLIAFSSYNKFTNNCYRDALVTTAINSLTSFSSGFVVFSFLGYMAQKH

SVPIRDVAKDGPGLIFVIYPEAIATLPLSSAWAVLFFTMLLALGIDSAVS

DRGPEPHGLCTAF

>moTH

MPTPSASSPQPKGFRRAVSEQDAKQVEAIMSPRFIGRRQSLIED

ARKEREAAAAAAAAAAVASSEPGNPLESVVFEEQDGMAILNLLFSLKGTKPSSLSRAV

KVFETFEAKIHHLETRPAQRPLAGSPHLEYFVRFEVPSGDLAALLSSIRRVSDDVRSA

REDKVPWFPRKVSELDKCHHLVTKFDPDLDLDHPGFSDQVYRQRRKLIAEIAFQYKQG

EPIPHVEYTAEEIATWKEVYATLKGLYATHACREHLEAFQLLERYCGYREDSIPQLED

VSRFLKERTGFQLRPVAGLLSARDFLASLAFRVFQCTQYIRHASSPMHSPEPDCCHEL

LGHVPMLADRTFAQFSQDIGLASLGASDEEIEKLSTVYWFTVEFGLCKQNGELKAYGA

GLLSSYGELLHSLSEEPEVRAFDPDAAAVQPYQDQTYQPVYFVSESFSDAKDKLRNYA

SRIQRPFSVKFDPYTLAIDVLDNPHTIRRSLEGVQDELHTLTHALSVIS

>mmuTH sp|P24529|TY3H_MOUSE Tyrosine 3-monooxygenase OS=Mus musculus GN=Th PE=1 SV=3

MPTPSASSPQPKGFRRAVSEQDTKQAEAVTSPRFIGRRQSLIEDARKEREAAAAAAAAAV

ASAEPGNPLEAVVFEERDGNAVLNLLFSLRGTKPSSLSRALKVFETFEAKIHHLETRPAQ

RPLAGSPHLEYFVRFEVPSGDLAALLSSVRRVSDDVRSAREDKVPWFPRKVSELDKCHHL

VTKFDPDLDLDHPGFSDQAYRQRRKLIAEIAFQYKQGEPIPHVEYTKEEIATWKEVYATL

KGLYATHACREHLEAFQLLERYCGYREDSIPQLEDVSHFLKERTGFQLRPVAGLLSARDF

LASLAFRVFQCTQYIRHASSPMHSPEPDCCHELLGHVPMLADRTFAQFSQDIGLASLGAS

DEEIEKLSTVYWFTVEFGLCKQNGELKAYGAGLLSSYGELLHSLSEEPEVRAFDPDTAAV

QPYQDQTYQPVYFVSESFSDAKDKLRNYASRIQRPFSVKFDPYTLAIDVLDSPHTIRRSL

EGVQDELHTLTQALSAIS

>rnTH ENSRNOT00000027682 length=498

MPTPSAPSPQPKGFRRAVSEQDAKQAEAVTSPRFIGRRQSLIEDARKERE

AAAAAAAAAVASSEPGNPLEAVVFEERDGNAVLNLLFSLRGTKPSSLSRA

VKVFETFEAKIHHLETRPAQRPLAGSPHLEYFVRFEVPSGDLAALLSSVR

RVSDDVRSAREDKVPWFPRKVSELDKCHHLVTKFDPDLDLDHPGFSDQVY

RQRRKLIAEIAFQYKHGEPIPHVEYTAEEIATWKEVYVTLKGLYATHACR

EHLEGFQLLERYCGYREDSIPQLEDVSRFLKERTGFQLRPVAGLLSARDF

LASLAFRVFQCTQYIRHASSPMHSPEPDCCHELLGHVPMLADRTFAQFSQ

DIGLASLGASDEEIEKLSTVYWFTVEFGLCKQNGELKAYGAGLLSSYGEL

LHSLSEEPEVRAFDPDTAAVQPYQDQTYQPVYFVSESFNDAKDKLRNYAS

RIQRPFSVKFDPYTLAIDVLDSPHTIQRSLEGVQDELHTLAHALSAIS

>cpTH ENSCPOT00000012712 length=498

MPTPSTAAPQPKGFRRAVSELDAKQAEAIMSPRFIGRRQSLIEDARKERE

AAEAAAAAAVASSEPGDPLEAVVFEEKEGRAVLNLLFSLRGSKPSPLSRA

VKVFETFEAKIHHLETRPAPRPQAGGPCLEYFVRFEVPRGDLPALLSSVR

RVSDDVRSPKEDKVPWFPQKVAELDKCHHLVTKFEPDLDLDHPGFSDQVY

RQRRKLIAEIAFQYKHGEPIPLVEYTAEEIATWKEVYTTLKGLYATHACR

EHLEAFQLLERFCGYREDSIPQLEDVSRFLKERTGFQLRPVAGLLSARDF

LASLAFRVFQCTQYIRHASSPMHSPEPDCCHELLGHVPMLADRTFAQFSQ

DIGLASLGASDEEIEKLSTLYWFTVEFGLCKQNGELKAYGAGLLSSYGEL

LHSLSEEPEIRAFDPEAAAVQPYQDQTYQSVYFVSESFSDAKAKLRSYAS

RIQRPFSVKFDPYTLAIDVLDNPHAIQRSLEGVQDELHTLVHALGAIS

>ocTH gi|217418319|gb|ACK44320.1| tyrosine hydroxylase isoform b (predicted) [Oryctolagus cuniculus]

MPTPNAPAPQARGFRRAVSELDAKQVEAVMSPRFVGRRQSLIEDARKEREAAAAAAAAAALEAGDPLEKL

SCQERAGQAVLSLLFSLRDARPSLSRAVKVFESFEAKIQHLETRPAPGRRAGSPHLECFVRCEAPRGDLA

ALLSSLRRVTEDVRSAGDHKVPWFPRRVAELDKCHHLVTKFDPDLDLDHPGFSDQAYRQRRKQIADIAFQ

YKHGSPIPRVEYTAEEIATWKEVYSTLRGLYATHACREYLQALELLERCSGYSEDHIPQLEDVSRFLKER

TGFQLRPVAGLLSARDFLASLAFRVFQCTQYIRHASSPMHSPEPDCCHELLGHVPMLADRTFAQFSQDIG

LASLGASDEAIEKLSTLYWFTVEFGLCKQNGEVKAYGAGLLSSYGELLHSLSEEPEIRAFDPDAAAVQPY

QDQTYQPVYFVSESFSDAKNKLRSYASRIQRPFSVKFDPYTLAVDVLDSPQAIGRSLEDVQDELHTLAQA

LSTLG

>moUCN

MRQRGRAALLVALLLLAQLRPGSSQWSPVTAAATEVQDPNLRWSPGTRNQGGGARALLLLLAERFPRRTGSGTPGGERQ

RRDDPPLSIDLTFHLLRTLLELARTQSQRERAEQNRIILHSVGK

>mmuUCN ENSMUST00000043475 length=122

MIQRGRATLLVALLLLAQLRPESSQWSPAAAAATGVQDPNLRWSPGVRNQGGGVRALLLLLAERFPRRAGSEPAGERQR

RDDPPLSIDLTFHLLRTLLELARTQSQRERAEQNRIIFDSVGK

>rnUCN ENSRNOT00000008037 length=122

MRQRGRATLLVALLLLVQLRPESSQWSPAAAAANVVQDPNLRWNPGVRNQGGGVRALLLLLAERFPRRAGSEPAGERQR

RDDPPLSIDLTFHLLRTLLELARTQSQRERAEQNRIIFDSVGK

>ocUCN gi|291387031|ref|XP_002710002.1| PREDICTED: urocortin-like [Oryctolagus cuniculus]

MRQAGRAALLGALLLLAQLRPGSSQWSPAEAEAAAGVQDPSLRWSPGTRNHGGGARALLLLLAERFPRRAGSGSWGPRT

ASERPRRDDPPLSIDLTFHLLRTLLELARTQSQRERAEQNRVIFDSVGK

>moUCN3

MLMPTYFLLLLLLLLGSPGTSLSRKFYNTGPVFSCLNMALSEVK

KNKLEDVPLLSKNSFHHLPPQDSLGEEEEKQKHGKSKRTFSGPAGGSGAGSLRYRYQS

QAQHKGKLYQDKVKSDRGTKFTLSLDVPTNIMNILFNIDKAKNLRAKAAANAQLMAQI

GKK

>mmuUCN3 NP_112540 length=164

MLMPTYFLLPLLLLLGGPRTSLSHKFYNTGPVFSCLNTALSEVKKNKLED

VPLLSKKSFGHLPTQDPSGEEDDNQTHLQIKRTFSGAAGGNGAGSTRYRY

QSQAQHKGKLYPDKPKSDRGTKFTLSLDVPTNIMNILFNIDKAKNLRAKA

AANAQLMAQIGKKK

>rnUCN3 NP_001073677 length=164

MLMPTYFLLLLLLLLGGPRTSLSHKFYNAGPVFSCLNTALSEVKKNKLED

VPVLSKKNFGYLPTQDPSGEEEDEQKHIKNKRTFSDAVGGNGGRSIRYRY

QSQAQPKGKLYPDKVKNDRGTKFTLSLDVPTNIMNILFNIDKAKNLRAKA

AANAQLMAQIGKKK

>cpUCN3 ENSCPOT00000027240 length=167

MLMPAHFWLLLILLLGDSSTSLSHKFCKAGSVFNCLNTALCKVQKNQRED

VPLLSKNSFHYLPSQDLSFREEKEEEKENLDKDKRTFPDSGGGGGAGSTR

YKYLPQAQHKGKLYQDKGKSDQSTKFTLSLDVPTNIMNILFKIAKVKNMQ

AKAAANAHLMAQIGRKK

>ocUCN3 ENSOCUT00000013769 length=166

LIKSTKLISLVILKAGPRTGLSHKFYKDKPIFSCLNTALSEAKKSQLEAV

SLLSKRSFLYLPSQGPSSGEEEKEREEKQDKEKRTSPGSGGSSGAGSIRY

KYLSQAQLRGKVYQDKAKSDRHTKFTLSLDVPTNIMNILFNIAKAKNLQA

KAAANAHLMAQIGRKK
