# Supplementary material for: BAC-Based Sequencing of Behaviorally-Relevant Genes in the Prairie Vole
Source: PLoS One. 2012 Jan 6;7(1):e29345. doi: 10.1371/journal.pone.0029345 (PMC3253076; doi:10.1371/journal.pone.0029345)
Supplement: Table S1 — GenBank accession numbers for assembled and annotated prairie vole sequences. (DOC) [file pone.0029345.s002.doc]

| **Table S1.** GenBank accession numbers for assembled and annotated prairie vole sequences. | | |  |
| --- | --- | --- | --- |
| Target gene(s) | GenBank Ac.# | |  |
| *Ar* | | DP001206.1 | |
| *Avpr1a* | | DP001225.1 | |
| *Avpr1a* Truncated | | HQ156469* | |
| *Bdnf* | | DP001207.1 | |
| *Crh* | | DP001210.1, DP001211.1 | |
| *Crhr1* | | DP001226.1, DP001227.1 | |
| *Crhr2* | | DP001213.1 | |
| *Drd1a* | | DP001231.1, DP001263.1 | |
| *Drd2* | | DP001219.1, DP001220.1 | |
| *Esr1* | | DP001223.1, DP001224.1 | |
| *Esr2* | | DP001228.1, DP001262.1 | |
| *Mc4r* | | HQ156468* | |
| *Nr3c1* | | DP001235.1, DP001236.1 | |
| *Ntrk2* | | DP001232.1 | |
| *Oxt, Avp* | | DP001208.1, DP001209.1 | |
| *Oxtr* | | DP001214.1, DP001215.2 | |
| *Slc6a2* | | DP001217.1, DP001218.1 | |
| *Slc6a3* | | DP001233.1, DP001234.1 | |
| *Th* | | DP001216.1 | |
| *Ucn* | | DP001212.1 | |
| *Ucn2* | | DP001221.1, DP001222.1 | |
| *Ucn3* | | DP001229.1, DP001230.1 | |

* Assemblies derived from sequencing a pool of clones using the 454 sequencing platform.
