# Supplementary material for: BAC-Based Sequencing of Behaviorally-Relevant Genes in the Prairie Vole
Source: PLoS One. 2012 Jan 6;7(1):e29345. doi: 10.1371/journal.pone.0029345 (PMC3253076; doi:10.1371/journal.pone.0029345)
Supplement: Table S2 — Amino acid substitutions specific to the prairie vole lineage. (DOC) [file pone.0029345.s003.doc]

| **Supplementary Table 2.** Amino acid substitutions specific to the prairie vole lineage | | |
| --- | --- | --- |
| Protein | Substitution | Type of amino acid substitution |
| AVP | T8S | Conservative |
| AVP | S38P | Conservative |
| AVP | G52D | Radical |
| AVP | S60N | Conservative |
| AVP | S134N | Conservative |
| AVP | A136G | Conservative |
| AVP | V151L | Conservative |
| AVP | L153M | Conservative |
| AVPR1A | G13S | Conservative |
| AVPR1A | T22S | Conservative |
| AVPR1A | G25D | Radical |
| AVPR1A | R30W | Radical |
| AVPR1A | G36Q | Conservative |
| AVPR1A | E37K | Radical |
| AVPR1A | D39S | Radical |
| AVPR1A | A164T | Conservative |
| AVPR1A | V180L | Conservative |
| AVPR1A | A264D | Radical |
| AVPR1A | G266A | Conservative |
| AVPR1A | E321D | Conservative |
| AVPR1A | S370R | Radical |
| AVPR1A | S382N | Conservative |
| AVPR1A | K411R | Conservative |
| AR | L7P | Conservative |
| AR | Q115H | Radical |
| AR | P141L | Conservative |
| AR | E182D | Conservative |
| AR | A224V | Conservative |
| AR | T289A | Conservative |
| AR | K325R | Conservative |
| AR | G329S | Conservative |
| AR | G333V | Conservative |
| AR | A359V | Conservative |
| AR | P385Q | Radical |
| AR | A428V | Conservative |
| AR | G460R | Radical |
| AR | A473V | Conservative |
| AR | R479H | Conservative |
| AR | T/A485V | Conservative |
| AR | G500T | Conservative |
| AR | P511S | Conservative |
| AR | Q648K | Radical |
| AR | S657T | Conservative |
| AR | H744Q | Radical |
| AR | T873A | Conservative |
| BDNF | F21L | Conservative |
| BDNF | E90G | Radical |
| BDNF | E91V | Radical |
| BDNF | R211K | Conservative |
| CRH | A9V | Conservative |
| CRH | V14M | Conservative |
| CRH | A131T | Conservative |
| CRHR1 | Q14H | Radical |
| CRHR1 | S147N | Conservative |
| CRHR1 | V312I | Conservative |
| CRHR1 | R349H | Conservative |
| CRHR1 | T383S | Conservative |
| CRHR2 | L7P | Conservative |
| CRHR2 | P56S | Conservative |
| CRHR2 | A75T | Conservative |
| CRHR2 | V263L | Conservative |
| CRHR2 | V300I | Conservative |
| DRD1A | E10G | Radical |
| DRD1A | G173D | Radical |
| DRD1A1 | D184A | Radical |
| DRD2 | K332R | Conservative |
| ESR1 | R62S | Conservative |
| ESR1 | E64G | Radical |
| ESR1 | T216A | Conservative |
| ESR2 | A253T | Conservative |
| ESR2 | R340G | Radical |
| ESR2 | S363P | Conservative |
| MC4R | A27T | Conservative |
| MC4R | A167G | Conservative |
| NR3C1 | G20V | Radical |
| NR3C1 | Y30H | Radical |
| NR3C1 | V39M | Conservative |
| NR3C1 | P46S | Conservative |
| NR3C1 | N137K | Radical |
| NR3C1 | T151P | Conservative |
| NR3C1 | E209Q | Conservative |
| NR3C1 | L246F | Conservative |
| NR3C1 | S264P | Conservative |
| NR3C1 | T336A | Conservative |
| NR3C1 | Q341E | Conservative |
| NR3C1 | Q343P | Radical |
| NR3C1 | T374A | Conservative |
| NR3C1 | S375N | Conservative |
| NR3C1 | G383A | Conservative |
| NR3C1 | V504A | Conservative |
| NR3C1 | S505A | Conservative |
| NR3C1 | T557N | Conservative |
| NR3C1 | G614A | Conservative |
| NR3C1 | D637E | Conservative |
| NR3C1 | H641Q | Radical |
| NR3C1 | S761T | Conservative |
| NTRK2 | L23V | Conservative |
| NTRK2 | R94K | Conservative |
| NTRK2 | I120V | Conservative |
| NTRK2 | I144V | Conservative |
| NTRK2 | S167G | Conservative |
| NTRK2 | K212N | Radical |
| NTRK2 | T288S | Conservative |
| OXT | S56N | Conservative |
| OXT | T112M | Radical |
| OXTR | T66I | Radical |
| SLC6A3 | R198K | Conservative |
| SLC6A3 | V227I | Conservative |
| TH | A72S | Conservative |
| TH | R78Q | Radical |
| TH | V83I | Conservative |
| TH | R91K | Conservative |
| TH | V150I | Conservative |
| TH | A497V | Conservative |
| UCN | A29V | Conservative |
| UCN | A69T | Conservative |
| UCN | A75G | Conservative |
| UCN | F118I | Conservative |
| UCN | D119H | Radical |
| UCN3 | G17S | Conservative |
| UCN3 | R/S19G | Conservative |
| UCN3 | H24R | Conservative |
| UCN3 | T38M | Radical |
| UCN3 | T/S64P | Conservative |
| UCN3 | S68L | Radical |
| UCN3 | A/S87P | Conservative |
| UCN3 | T/I96L | Conservative |

Location is based on the prairie vole protein sequences (longest) annotated in this study. The first amino acid listed represents the inferred ancestral state and the second is the amino acid observed in the prairie vole.
