# Supplementary material for: BAC-Based Sequencing of Behaviorally-Relevant Genes in the Prairie Vole
Source: PLoS One. 2012 Jan 6;7(1):e29345. doi: 10.1371/journal.pone.0029345 (PMC3253076; doi:10.1371/journal.pone.0029345)
Supplement: Table S3 — Nonsynonymous variants identified in the behaviorally-relevant prairie vole proteins. (DOC) [file pone.0029345.s004.doc]

| **Table S3** Nonsynonymous variants identified in the behaviorally-relevant prairie vole proteins | | | |
| --- | --- | --- | --- |
| Protein | Substitution | Type of amino acid substitution | SIFT |
| AVP | T116S | Conservative | Tolerated |
| AVP | R129S | Radical | Affect function |
| CRHR1 | V106A | Conservative | Affect function |
| CRHR1 | R113W | Radical | Affect function |
| CRHR1 | V202I | Conservative | Tolerated |
| DRD2 | A29V | Conservative | Tolerated |

Location is based on the prairie vole protein sequences (longest) annotated in this study.
